# Burden of epilepsy in Latin America and The Caribbean: a trend analysis of the Global Burden of Disease Study 1990 – 2019

**DOI:** 10.1016/j.lana.2021.100140

**Published:** 2021-12-16

**Authors:** Kevin Pacheco-Barrios, Alba Navarro-Flores, Alejandra Cardenas-Rojas, Paulo S. de Melo, Elif Uygur-Kucukseymen, Carlos Alva-Diaz, Felipe Fregni, Jorge G. Burneo

**Affiliations:** a SYNAPSIS Mental Health and Neurology Non-Profit organization, Research Department, Lima, Peru; b Neuromodulation Center and Center for Clinical Research Learning, Spaulding Rehabilitation Hospital and Massachusetts General Hospital, Harvard Medical School, Boston, MA, USA; c Universidad San Ignacio de Loyola, Vicerrectorado de Investigación, Unidad de Investigación para la Generación y Síntesis de Evidencias en Salud. Lima, Peru; d Georg-August-University Goettingen, International Max Planck Research School for Neurosciences, Goettingen, Germany.; e Grupo de Investigación Neurociencia, Efectividad Clínica y Salud Pública, Universidad Científica del Sur, Lima, Peru; f Department of Epidemiology, Harvard T.H. Chan School of Public Health, Boston, MA, USA.; g Neuro-Epidemiology Unit and Epilepsy Program, Department of Clinical Neurological Sciences, Western University, London, ON, Canada; h Kepez State Hospital, Kepez, Turkey; i Servicio de Neurología, Departamento de Medicina y Oficina de Apoyo a la Docencia e Investigación (OADI), Hospital Daniel Alcides Carrión, Callao, Peru

**Keywords:** Burden of disease, epilepsy, epidemiology (source: MESH)

## Abstract

**Background:**

The epilepsy prevalence in Latin America and the Caribbean (LAC) had remained high over the last 20 years. Data on the burden of epilepsy are needed for healthcare planning and resource allocation. However, no systematic analysis had been performed for epilepsy burden in LAC.

**Methods:**

We extracted data of all LAC countries from the Global Burden of Disease (GBD) study from 1990 to 2019. Epilepsy burden was measured as prevalence, mortality, and disability-adjusted life-years (DALYs; defined by the sum of years of life lost [YLLs] for premature mortality and years lived with disability [YLDs]), by age, sex, year, and country. Absolute numbers, rates, and 95% uncertainty intervals were reported. We performed correlational analyses among burden metrics and Socio-demographic Index (SDI).

**Findings:**

The burden of epilepsy decreased around 20% in LAC, led by YLLs reduction. In 2019, 6·3 million people were living with active epilepsy of all causes (95% UI 5·3 - 7·4), with 3·22 million (95% UI 2·21 - 4·03) and 3·11 million (95% UI 2·21 to 4·03) cases of epilepsy with identifiable aetiology and idiopathic epilepsy, respectively. The number of DALYs represented the 9·51% (1.37 million, 95% UI 0·99 -1·86) of the global epilepsy burden in 2019. The age-standardized burden was 175·9 per 100 000 population (95% UI 119·4 - 253·3), which tend to have a bimodal age distribution (higher in the youth and elderly) and was driven by high YLDs estimates. The burden was higher in men and older adults, primarily due to high YLLs and mortality. Alcohol use was associated with 17% of the reported DALYs. The SDI estimates significantly influenced this burden (countries with high SDI have less epilepsy burden and mortality, but not prevalence or disability).

**Interpretation:**

The epilepsy burden has decreased in LAC over the past 30 years. Even though, LAC is still ranked as the third region with the highest global epilepsy burden. This reduction was higher in children, but burden and mortality increased for older adults. The epilepsy burden is disability predominant; however, the mortality-related estimates are still higher than in other regions. Alcohol consumption and countries’ development are important determinants of this burden. There is an urgent need to improve access to epilepsy care in LAC, particularly for older adults. Strengthening primary care with online learning and telemedicine tools, and promoting risk factors modification should be prioritized in the region.

**Funding:**

This research was self-funded by the authors.

**Research in context**

**Evidence before this study**

We searched in PubMed, using the keywords burden of disease [Title/Abstract] AND epilepsy [Title/Abstract] to identify published articles addressing the epilepsy burden in Latin America (LAC), without date or language restrictions (last search May 29, 2021). We also searched for systematic reviews on epilepsy in Latin America and the Caribbean (LAC) (Latin America [Title/Abstract] AND epilepsy [Title/Abstract]). We found 16 studies. Eleven studies addressed the epilepsy burden globally or focusing on other regions (seven of them assessed the global burden, and four studies reported the epilepsy burden in Europe, USA, and Kenya). Only one study addressed tangentially the epilepsy burden in LAC but without country-level analysis and using data until 2016. Besides, we found five systematic reviews exploring the epilepsy epidemiology in LAC, none of them estimated burden of disease metrics. Therefore, to date, no previous studies addressed the epilepsy burden in LAC, by country, age, sex, and socioeconomic status.

**Added value of this study**

This trend analysis of the Global Burden of diseases (GBD) study 2019 is aimed to provide an overview of the epilepsy impact in LAC over the past 30 years, and to identify gaps for further public health actions. We present regional and country-level results of prevalence, mortality, burden (disability-adjusted life-years [DALYs], years lived with disability [YLDs], and years of life lost [YLLs]), and attributable risk factors of all-cause active epilepsy, active idiopathic epilepsy (i.e., epilepsy of genetic or unknown origin), and epilepsy with identifiable etiology (only prevalence and YLDs). We also explore the influence of age, sex, and country's development status (socio-demographic index [SDI]) on the epilepsy burden in LAC countries.

**Implications of all the available evidence**

There was a significant reduction in the burden of epilepsy from 1990 to 2019 in LAC, driven by a decrease in premature death rates (YLLs). Even though, in 2019, LAC is ranked as the third region with the highest worldwide burden (9•5% of the global epilepsy burden). The current burden is disability predominant (YLDs), mainly in men compared to women, and with a bimodal age distribution (the youth and elderly). However, mortality and disability are increasing alarmingly in older adults compared to other regions. Besides, we found that 17% of DALYs are associated with alcohol consumption, and a higher country's SDI is associated with less burden due to premature deaths. These results suggest that further improvement can be made in the region by increasing the access to epilepsy care in low and middle SDI countries in LAC, strengthening primary care with online learning and telemedicine tools, and promoting risk factors modifications (reduction of alcohol abuse, sanitation improvement, healthy aging, and cardiovascular risk reduction), especially targeting older adults. Future GBD studies need to explicitly explore the specific burden of epilepsy by etiologies (e.g., neurocysticercosis, traumatic brain injury, stroke) that is currently assessed as one group, also there is a need for assessing the mortality and YLLs attributed to epilepsy with identifiable etiologies..

Alt-text: Unlabelled box

## Introduction

Epilepsy is a global health issue affecting patients without distinction of age, race, and socioeconomic class.^1^ By 2016, it was estimated that 45·9 million patients were living with active epilepsy worldwide.^2^ This condition is twice as common in low and middle-income countries as it is in the developed world.^2^ In Latin America and the Caribbean (LAC), the estimated lifetime and active epilepsy prevalence were 14·09 and 9·06 per 1,000 inhabitants respectively, while the incidence rate was 1·11 per 1,000 person-year,^3^ all values higher than the worldwide estimates.^4^

However, to understand a disease impact and its epidemiological behaviour, the classic prevalence and incidence estimates are insufficient. Other metrics are recommended to include not only the disease frequency but also the degree of health loss produced by the disease (considering deaths and residual disability).^5^ The Global Burden of Disease (GBD) study is an example of this approach. They estimated several metrics such as disability-adjusted life year (DALY), which is the sum of the years of life lost (YLL) and the years lived with disability (YLD) due to certain conditions; mortality rates; and risk factors.^6^

According to the 2016 GBD study, the global age-standardized epilepsy burden (DALYs) were 182·6, the YLDs were 102·6, and the total amount of epilepsy-related deaths worldwide was estimated as 126 055.^2^ The epilepsy burden was higher in men, people in the extremes of life, and countries with low socio-demographic index (SDI). ^2^This report discussed their findings at a regional level; thus, it did not present a comprehensive country-level analysis of low- and middle-SDI regions to further characterize the factors and trends associated with this high burden.

While the GBD report encourages a differential analysis by regions and countries,^7^ the available information on the GBD platform does not allow for further visualization of the LAC region with calculations for changes and trends over time and neither changes by sex and age groups, which affects the data usability for regional prioritization and health policies. Furthermore, the complex interplay of health determinants in LAC underscores the need for epilepsy burden assessment to assure an adequate economic, clinical, and scientific resources allocation in the region. Thus, we aimed to conduct a trend epidemiological profiling of epilepsy in LAC using the data provided by the GBD study from 1990 to 2019.

## Methods

### The Global Burden of Disease Study 2019

The methodology of the GBD Study 2019 had been described elsewhere.^8^^,^^9^ In summary, the GDB Study group estimates the burden of disease by performing a Bayesian meta-regression modelling (using the DisMod-MR 2·1 software). The modelling is based on i) available data (population surveys and systematic reviews with meta-analysis) from countries worldwide, and ii) predictive estimations for countries with sparse or no data. The metrics calculated are adjusted for possible confounders (exposure measure of alcohol consumption and pig meat consumption per capita). The provided metrics are prevalence, mortality, burden metrics (Disability-adjust life years [DALYs], years of life lost [YLLs], and years lived with disability [YLDs]), and risk factors.^9^ Additionally, they reported an indicator of the country's socio-economic development called socio-demographic Index (SDI), this indicator is a composite of the income per capita, the years of school education, and the fertility rate in women younger than 25 years old. ^9^

### Latin America and the Caribbean countries

For this analysis, we utilized data from 17 individual countries that belong to Latin America and the Caribbean region.^10^ We included the following five sub-regions proposed by the GBD study^11^: a) Andean Latin America (Bolivia, Ecuador y Peru), b) Caribbean (all islands, including Puerto Rico – we extracted the whole sub-region since the data of individual countries was incomplete), c) Central Latin America (Colombia, Costa Rica, El Salvador, Guatemala, Honduras, Mexico, Nicaragua, Panama and Venezuela), d) Tropical Latin America (Brazil, Paraguay), e) Southern Latin America (Argentina, Chile, and Uruguay).

### Epilepsy definitions

Similar to the global report for epilepsy,^2^ we report the GBD study data for overall epilepsy, which includes the idiopathic and secondary components. For overall epilepsy, the case definition used by the GBD study was based on the International League Against Epilepsy (ILAE) Guidelines for Epidemiologic Studies on Epilepsy: two or more unprovoked recurrent seizures with at least one occurring in the last five years, with or without treatment.^12^ The diagnostic codes used for epilepsy were 345 of the ICD-9, and G40 and G41 of the ICD-10.^13^^,^^2^

Idiopathic active epilepsy was defined by the GBD study according to the 1985 ILAE proposal,^14^ and included those of genetic or unknown causes.

Data on epilepsy with identifiable aetiology data was calculated as the subtraction of idiopathic epilepsy from the overall epilepsy estimate.^2^ However, the GBD study provided the complete burden metrics only for idiopathic epilepsy; thus, the main analysis focused on the idiopathic subgroup.

### GBD study metrics definitions

Disability-adjust life years (DALYs): This metric is calculated as: “the sum of the years of life lost because of premature mortality (YLL) and the years of healthy life lost due to disability.”^6^

Years of life lost (YLLs): This metric is calculated as the number of deaths per age group multiplied for the remaining years to live according to countries’ life expectancy based on the GBD standard life table.^15^

Years lived with disability (YLDs): This is calculated as: “the number of incident cases in a certain period of time multiplied by the average of disease duration and by a weight factor that reflects its severity which ranges from 0 (perfect health) to 1 (deceased).”^6^

Deaths: The cause of death is attributed to epilepsy if in the death certificate the ICD-10 codes G40 or G41 were reported as the cause of death either confirmed or suspected. The exact mechanism was not available (e.g., status epilepticus, accidents associated with seizures, or sudden unexpected death in epilepsy [SUDEP]).

Risk Factors: The methodology for the calculation of risk factors is explained in detail elsewhere.^16^ In summary, the population-attributable fraction (expressed in percentage) was estimated using data for exposure, relative risk, and a theoretical-minimum exposure level. This metric is interpreted as the degree of burden explained by a certain risk factor. For epilepsy, 87 risk factors were explored, but they only reported results that showed an association.^17^

### Data extraction procedure

Data was extracted independently by two researchers from the GBD 2019 database for global and Latin America and the Caribbean region, including a total of 17 countries and five subregions, from 1990 to 2019. We extracted epilepsy data from two contexts: “impairment” (for overall epilepsy and epilepsy with identifiable aetiology) and “cause” (for idiopathic epilepsy). We extracted six metrics per country: SDI, prevalence, mortality, DALYs, YLLs, and YLDs with their corresponding 95% uncertainty intervals. The metrics were expressed as absolute numbers, and relative rates (all ages and age-standardized rates by 100 000 inhabitants. Besides, we extracted the burden data attributable to risk factors (the percentage of DALYs associated with a specific risk factor): from the 87 risk factors assessed in the GBD 2019 study, the only risk factor associated with epilepsy burden was alcohol use, hence, it was the only value for extraction. Finally, we extracted the aforementioned metrics divided into sub-groups based on age (under 5, 5 to 14, 15 to 49, 50 to 69, and more than 70 years old) and sex (female and male). The datasets were requested and downloaded from the open-access GBD study website: http://ghdx.healthdata.org/gbd-results-tool. The data visualization options available in the GBD platform are multiple. However, an option to evaluate trends and changes over time for the complete LAC region, and by age groups and sex is currently not provided on the website. The data was tabulated and organized in a Microsoft Excel spreadsheet.

### Statistical analysis

For the descriptive analysis of variables, the absolute numbers, rates and their 95% uncertainty interval were extracted and used directly from the GBD platform. For estimates using numbers, the aggregated geographical data (LAC overall and subregions) was calculated by arithmetic summing the countries’ values, while for age-standardized rates, the weighted arithmetic mean was used. We calculated the additional estimates percent of change from 1990 to 2019 to compare the overall trends across sub-regions and countries that were not originally provided in the platform. We manually calculated the percentage of change by using an arithmetic “rule of three”. Also, the annualized rate of change from 1990 to 2019 was calculated by subtracting the values from 2019 and 1990, then dividing it by the number of years (30 years). In both calculations, the formulas were applied for both the estimates in number and rates using the data from the GBD study. Moreover, we performed an exploratory correlational analysis among burden metrics and the countries’ SDIs using Spearman's rank correlation test since we expected non-normal aggregate data. We considered a 0·05 significance level for all the analyses. Analyses were conducted using R version 4·0·2.^18^

### Role of the funding source

This study was self-funded by the authors. No external source had any role in the study design, data collection, data analysis, interpretation of the results or writing of the report.

## Results

### Prevalence

Our results indicated that in 2019, there were 6,342,165·7 (95% UI 5,318,849 to 7,415,361·3) individuals with all-cause epilepsy in LAC, and the age-standardized rate was 817 (95% UI; 649·7 - 976·8). A similar number of cases were attributed to idiopathic cases (49%) and cases with identifiable aetiology (51%). For idiopathic epilepsy, there were 3,116,748·4 patients, which represented an increase of 44·3% from 1990. For epilepsy with identifiable etiology, there were 3,225,417·3 cases (95% UI 2,218,511·1 - 4,039,169·3), which meant an increase of 107·8% from 1990. This prevalence in LAC represented 8·1% of the global prevalence in 2019. The age-standardized prevalence rate (per 100 000 population) of epilepsy with identifiable aetiology in 2019 was 1222·1 (95% UI, 392·10 to 416·8), which increased from 1990 (10·8%). Moreover, the age-standardized prevalence rate (per 100 000 population) of idiopathic epilepsy in 2019 was 411·9 (95% UI, 257·6 to 560), with virtually no changes since 1990 (-0·0003%). The prevalence rate was similar in males (410; 95% UI 257·9 to 555·9) and females (413·70; 9% UI, 256·9 to 565·9). The higher prevalence was found in patients of 70 years and older (664·5; 95% UI, 413·2 to 925·4), followed by the 5- to 14-year-old group (419·4; 95% UI, 229·5 to 652·5), and the 50- to 69-year-old age group (409; 95% UI, 244·4 to 581·8).

The region with the highest prevalence rate for all LAC in 2019 was Central Latin America (573·4, 95% UI, 410·7 to 752), the region with the lowest prevalence rate was Southern Latin America with 327·9 (95% UI, 153·9 to 484·3). Paradoxically, the region with the highest increment from 1990 to 2019 was Southern Latin America (+10·7%), the region with the highest decrement was Tropical Latin America (-13·7%).

The country with the lowest prevalence rate in 2019 was Argentina with 267·9 (95% UI, 83·7 to 428·9) followed by Uruguay 381·1 (95% UI, 106·9 to 612·2), the country with highest prevalence rate was Ecuador 659·7 (95% UI, 177·4 to 1089·2). The country with the most incremental change was Panama (+30·8%), followed by Uruguay (18·5%). The countries with the highest decrement were Bolivia (-20·9%), and Brazil (-14·2%).

However, the differences by age or geographical location were not statistically significant due to overlapping confidence intervals.

### Mortality

**Figure 1:**
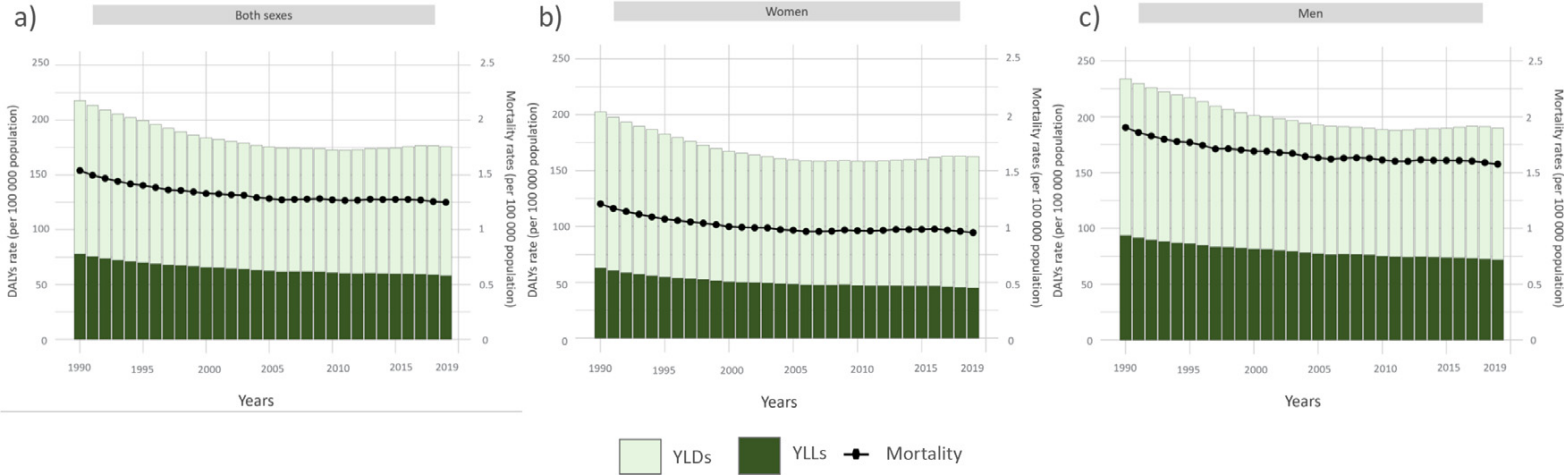
Age standardized DALYs, YLLs, YLDs and mortality rates (per 100 000 population) in Latin America and the Caribbean from 1990 to 2019. Both sexes (a), women (b), and (c) men.

DALYs, Disability-adjusted life years; YLLs, years of life lost; YLDs, years lived with disability.

No data on all-cause epilepsy mortality were available; thus, we calculated estimates only for idiopathic epilepsy. The age-standardized mortality rate (per 100 000 population) due to active idiopathic epilepsy in LAC in 2019 was 1·25 (95% UI, 1·1 to 1·4), a 19% less since 1990. It has decreased constantly since 1990 until around 2002 and since then has remained steady until 2019. Changes in mortality rates over the years from 1990 to 2019 are presented in Figure 1. In 2019, it was higher in men (1·6; 95% UI, 1·4 to 1·7) than in women (0·9; 95% UI, 0·8 to 1·1), similarly that in 1990 **(**Figure 1**)**.

**Figure 2:**
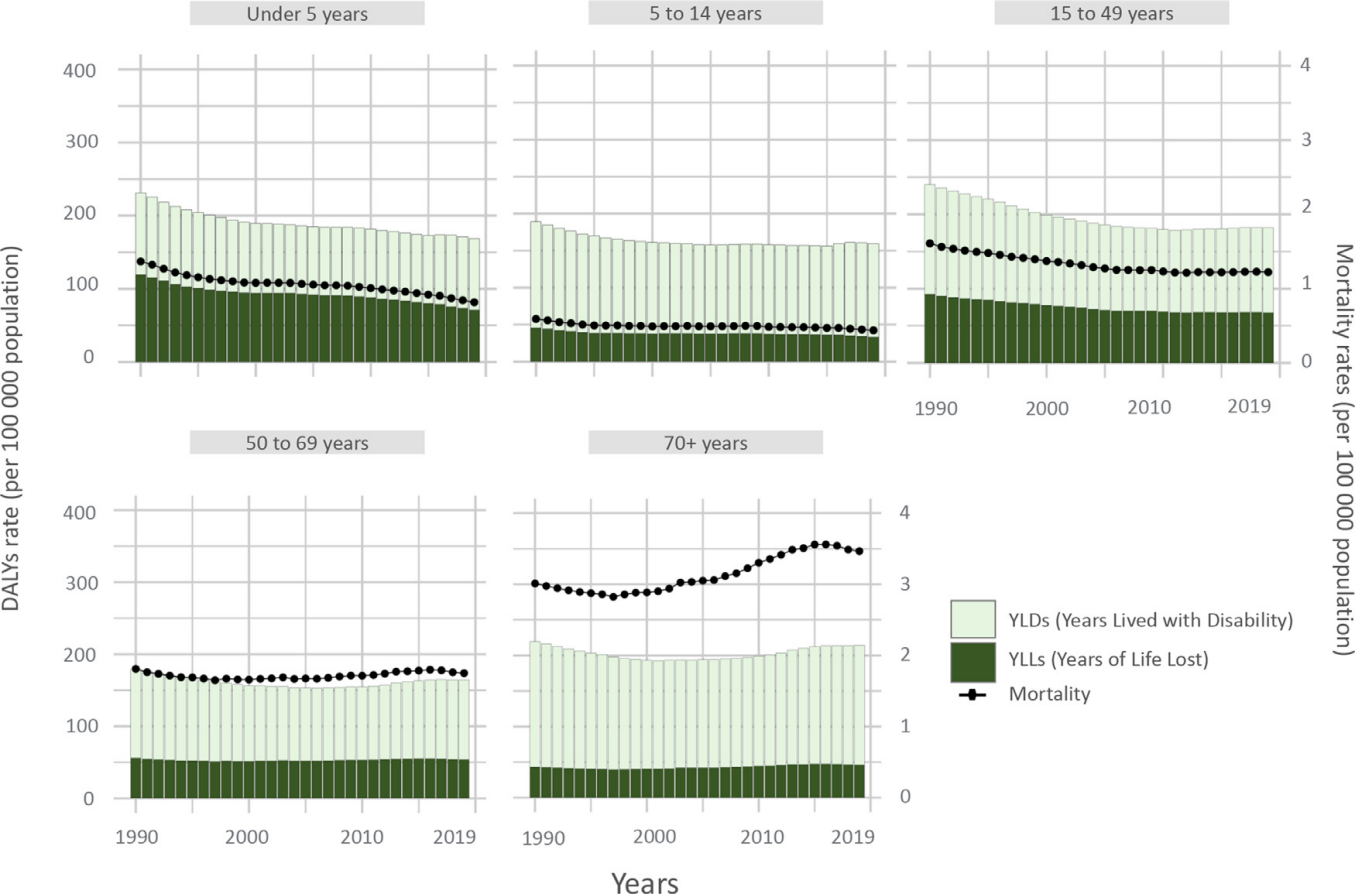
DALYs, YLLs, YLDs and mortality rates (per 100 000 population) by age subgroup in Latin America and the Caribbean from 1990 to 2019.

DALYs, Disability-adjusted life years; YLLs, years of life lost; YLDs, years lived with disability.

From 1990 to 2019, it had decreased around 50% in children under 5 years old. It had remained still in the age group of 5 to 14 years old, 15- to 49-year-old, and in the group of 50 to 69 years old. It had a bimodal increment in the 70+ group, it initially decreased from 1990 to 2000, and then increased progressively until 2019. The progress of mortality rates by years and by age groups is presented in Figure 2.

The region with the highest age-standardized mortality rate was the Caribbean (2.1; 95% UI, 1.6 to 2·6) and the one with the lowest was Southern Latin America (0·9; 95% UI, 0·9 to 1). The country with the highest mortality rate was Honduras (3·2; 95% UI, 2·3 to 4·4) followed by Guatemala (2·7; 95% UI, 2·2 to 3·5). The country with the lowest mortality rate was Argentina (0·7; 95% UI, 0·7 to 0·8), followed by Peru (0·8; 95% UI, 0·6 to 1·1). The differences between countries with the highest and lowest mortality rates were statistically significant by means of confidence intervals.

### Burden of Epilepsy

#### DALYs

We did not calculate DALYs for all-cause epilepsy and epilepsy with identifiable aetiology since YLLs estimates were not available, hence, we estimated DALYs only for idiopathic epilepsy. The total number of DALYs for idiopathic epilepsy in 2019 was 1,375,066·9 (95% UI, 997,125·3 to 1,861,732·3), which meant an increase of 13·6% from 1990. It represented 9·5% of the total world DALYS in 2019. The age-standardized rate of DALYs in LAC was 175·9 (95% UI, 119·4 to 253·3), a 19·3% less than in 1990. The age-standardized burden was higher in men (190; 9% UI, 133·8 to 266·3) than in females (162·57; 9% UI, 105·8 to 239·9) in 2019, as it was in 1990 (Figure 1).

**Figure 3:**
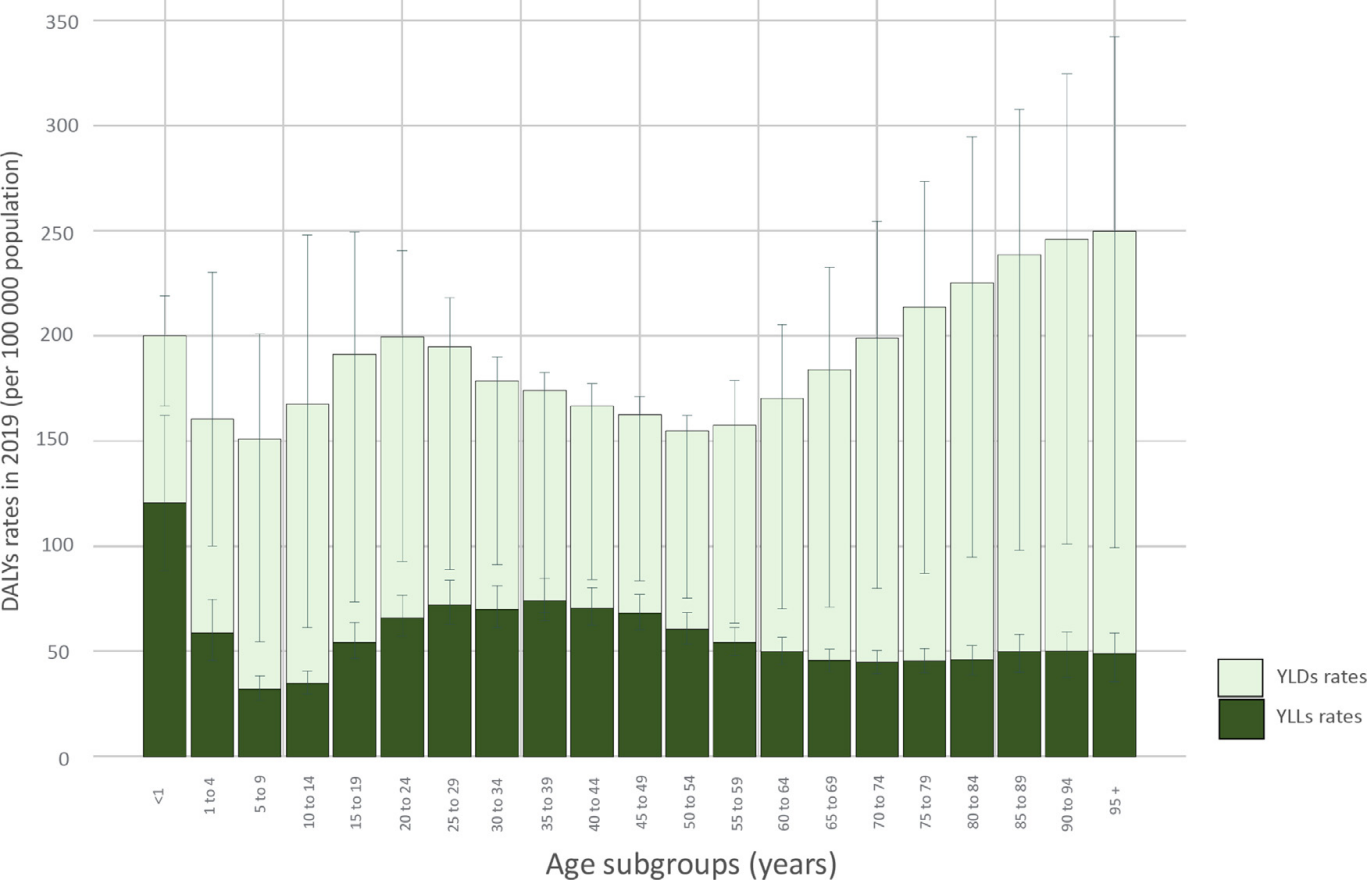
Trend of DALYs, YLDs, and YLLs rates (per 100 000 population) due to idiopathic epilepsy in LAC across age subgroups, 2019.

YLLs, years of life lost; YLDs, years lived with disability. Error bars represent UI 95%.

In the under 5 years-old groups, DALYs had decreased by 27%, and both YLDs and YLLs components contributed equally. DALYs were the highest in the 70+ group, with an 80% component of YLDs (Figure 2). The rest of the age groups remain still and high (around 200 DALYs per 100 000 population) across time. In 2019, the DALYs and its components have a multimodal behaviour across the lifespan. YLLs peaked at age under 5 years and had a plateau at 25 to 49 years old, then decreased progressively till 70 years old, then again has a slow increase until 95 plus years old (Figure 3). YLDs peaked at 15 to 19 years of age, decreased until 40–49 years, and increased exponentially to the oldest age group (> 60 years old), this second peak is almost double compared with younger ages groups (Figure 3).

**Figure 4:**
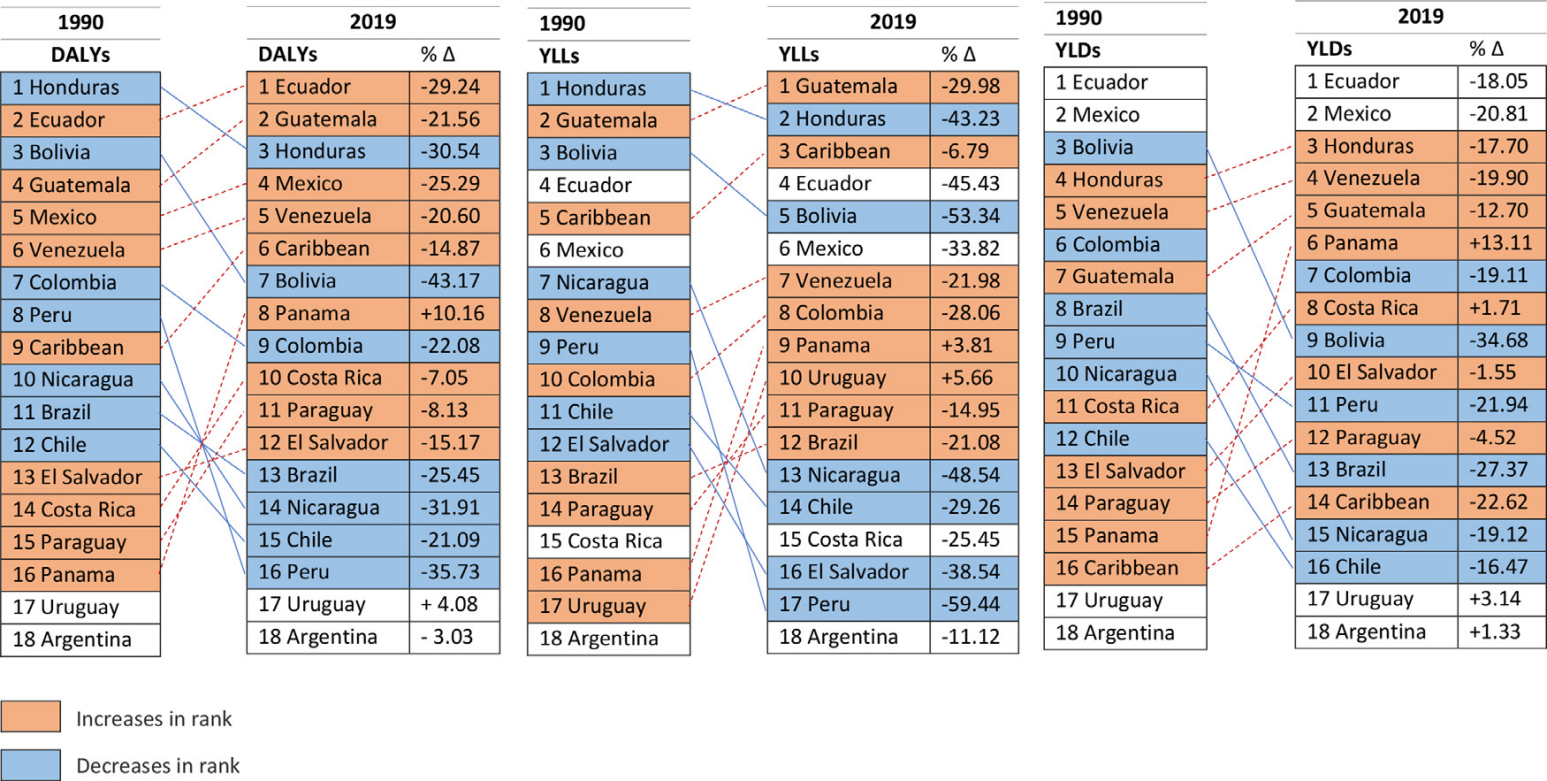
Burden of epilepsy by Country Rank by aged-standardized rates.

**Figure 5:**
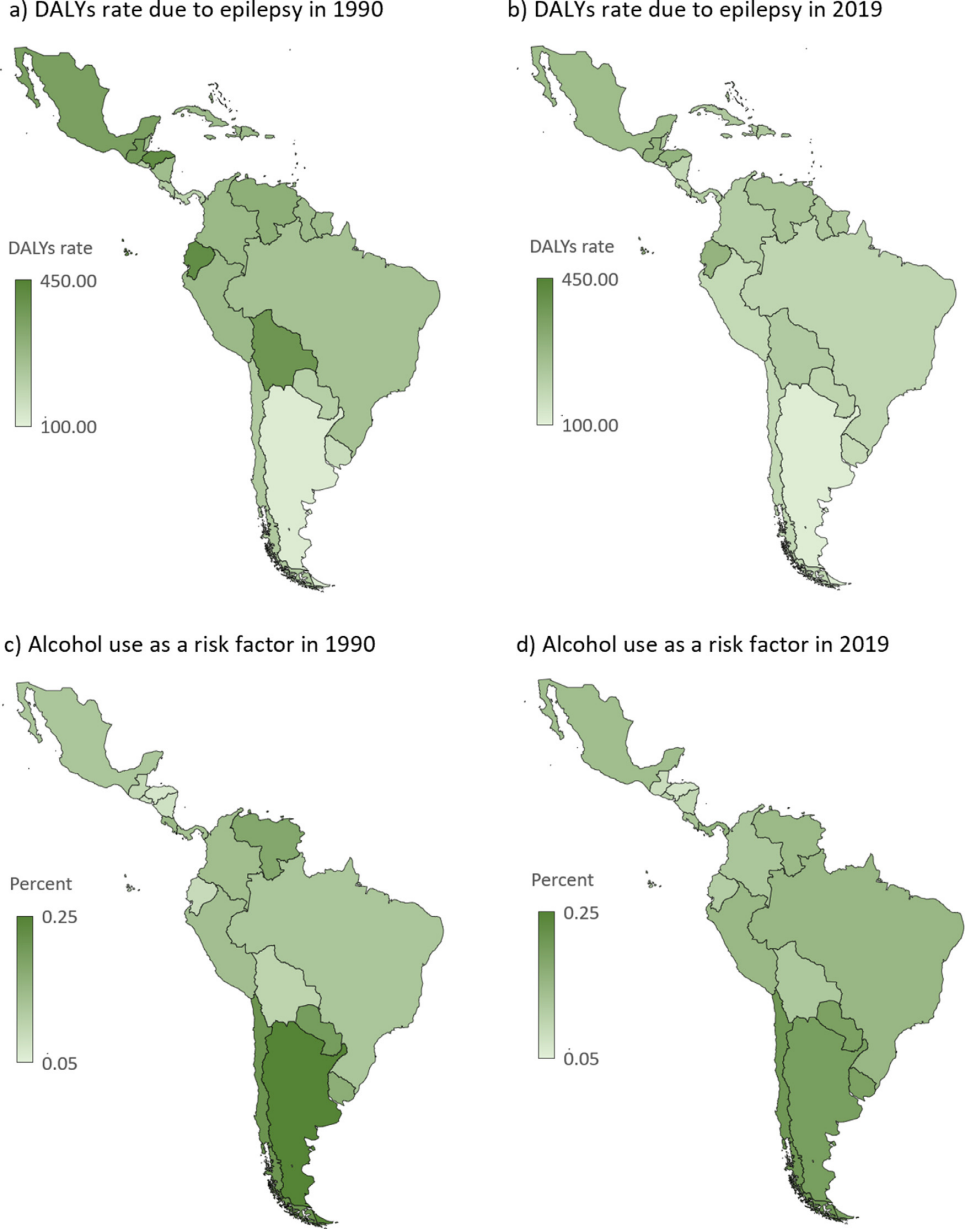
Geographical assessment: a, b) Age-standardized DALYs rates (per 100 000 population) in Latin America and the Caribbean in 1990 (a) and 2019 (b). c, d) Alcohol use as risk factor of epilepsy burden (age-standardized) in LAC in 1990 (c) and 2019 (d). The risk factor impact is presented as the percent of associated DALYs.

The region with the higher age-standardized rate was Central Latin America (249·5; 95% UI, 178·6 to 339·5); while the region with the lowest rate was Southern Latin America (131·9; 95% UI, 77·7 to 211·5). The country with the highest age-standardized rate of DALYs was Ecuador (293·2; 95% UI, 142·1 to 496·3) followed by Guatemala (292·5; 95% UI, 164·5 to 465·7). The countries with the lowest rate of DALYs were Argentina (110·9; 95% UI, 56·9 to 197·7) and Uruguay (167·7; 95% UI, 84·9 to 291·9) (Figure 4). Those geographical differences presented overlapping confidence intervals. The total DALYs by country in 1990 and 2019 are presented in Figure 5.

DALYs, Disability-adjusted life years.

#### YLDs

For all-cause epilepsy, the total number of YLDs was 2,102,737·4 (95% UI; 1,385,333·1 – 2,880,854·3) and the age-standardized rate was 268.6 (95% UI; 172·7 - 380·9) for LAC in 2019. Slightly higher numbers of YLDs were generated by epilepsy with identifiable aetiology (56·4%) compared to idiopathic cases (43·6%). For epilepsy with identifiable aetiology, the total number of YLDs in LAC in 2019 was 1,185,984·9 which meant a 110% increment from 1990. The age-standardized rate of YLDs was 419·3 (283·8 – 566·3), a 7·9% higher than the 1990 rate.

For idiopathic epilepsy, the total number of YLDs in LAC in 2019 was 916,752·5 which meant a 19·5% increment from 1990. Additionally, it represented 11·8% of the global YLDs due to epilepsy. The age-standardized rate of YLDs was 117·9 (61·6 – 195·5), a 15·8% lower than the 1990 rate.

The age-standardized rate of YLDs was similar in men (118·2; 95% UI, 62·3 to 194·7) and in women (117·7; 95% UI, 61·2 to 195·9). The age groups with a bigger decrement in YLDs were the 15- to 49-year-old group and in lesser account the 5-to-14-year group. The rest of the groups remained still through time.

The region with the higher rate was Central Latin America (168·7; 95% UI, 100·3 to 257·6); while the region with the lowest rate was Southern Latin America (88·7; 95% UI, 35·2 to 168·4). The country with the highest age-standardized rate of YLDs was Ecuador (200·8; 95% UI, 49·9 to 395·7) followed by Mexico (184·9; 95% UI, 108·1 to 276·5). The countries with the lowest rate of YLDs were Argentina (75·2; 95% UI, 21·3 to 161·6) and Uruguay (104·5; 95% UI, 20·7 to 226·8). However, those regional and by-country differences had overlapping confidence intervals (Figure 4).

#### YLLs

No YLLs data for all-cause epilepsy were available; thus, we calculated estimates only for idiopathic epilepsy. In 2019, the total number of YLLs for idiopathic epilepsy in LAC was 458,314·4, which meant an increment of 3·4% from 1990 and represented the 8·6% of the global YLLs due to epilepsy. The age-standardized rate of YLLs was 58 (95% UI, 51·9 to 64·9), that is a reduction of 25·5% from the 1990 rate. YLLs were higher in males (71·8; 95% UI, 64·5 to 80) than in females (44·9; 95% UI, 38.2 to 52·4). The biggest change in YLLs rate was found in the under 5 years in almost 50%. The smaller rate of YLLs was found in the 5- to 14-year-old group and in the 70+ group in both groups it had remained still.

The region with the higher rate was the Caribbean (106·3; 95% UI, 76·9 to 134) while the region with the lowest rate was Southern Latin America (43·2; 95% UI, 39·6 to 46·3). The country with the highest age-standardized rate of YLLs was Guatemala (133·8; 95% UI, 104·5 to 171·3) followed by Honduras (118·7; 95% UI, 79·9 to 167·6). The countries with the lowest rate of YLLs were Argentina (35·6; 95% UI, 31·9 to 38·3) and Peru (40·5; 95% UI, 28·6 to 56·1). However, differences between countries had overlapping confidence intervals (Figure 4).

A summary of all the estimates by country and their percentages of change from 1990 to 2019 is presented in Table 1.

**Table 1:** Burden of idiopathic epilepsy in Latin America by country, in absolute numbers and in age-standardized rates for 1990 and 2019, in both sexes.

	1990		2019		Changes			
	Number (in thousands) - (95% UI)	Age standardized rate (by 100 000 population) - (95% UI)	Number (in thousands) - (95% UI)	Age standardized rate (by 100 000 population) - (95% UI)	Percentage change for numbers - (95% UI)	Annualised rate of change, 1990–2019 (in numbers)	Percentage change for rates - (95% UI)	Annualised rate of change, 1990–2019 (in rates)
Global								
*Prevalence*	15324·1 (11446·7 - 19630·6)	288·8 (218·2 - 364·8)	25111·1 (19033·6 - 31433·0)	326·3 (247·8 - 408·3)	63·9 (60·1 - 66·3)	326·2 (252·9 - 393·4)	13·0 (11·9 - 13·6)	1·2 (1·0 - 1·5)
*Deaths*	100·1 (81·2 - 112·2)	1·9 (1·6 - 2·2)	114·0 (100·2 - 129·9)	1·5 (1·3 - 1·7)	13·9 (10·4 - 15·8)	0·5 (0·2 - 0·6)	-24·8 (-30·7 - -12·4)	0·0 (-0·1 - 0·2)
*DALYs*	11285·6 (8614·1 - 14136·6)	204·3 (157·6 - 254·1)	13077·6 (9986·7 - 16734·1)	170·6 (130·4 - 218·3)	15·9 (12·9 - 18·4)	59·7 (45·8 - 86·6)	-16·5 (-17·3 - -14·1)	-1·1 (-3·1 - -0·2)
*YLLs*	5897·0 (4542·8 - 6774·0)	104·6 (81·6 - 119·2)	5336·8 (4722·7 - 6170·1)	69·5 (61·7 - 80·6)	-9·5 (-18·0 - 4·0)	-18·7 (-20·1 - 6·0)	-33·6 (-44·4 - -22·4)	-1·2 (-2·7 - -0·3)
*YLDs*	5388·6 (3355·7 - 7974·0)	99·7 (63·1 - 146·1)	7740·8 (4810·3 - 11216·7)	101·1 (63·1 - 146·8)	43·7 (40·7 - 48·3)	78·4 (48·5 - 108·1)	1·4 (0·0 - 2·5)	0·0 (-0·2 - 0·3)
Overall Latin America								
*Prevalence*	2159·3 (1519·7 - 2872·8)	411·9 (268·6 - 563·8)	3116·8 (2218·5 - 4039·1)	411·9 (257·6 - 560·0)	44·3 (40·6 - 46·0)	31·9 (23·3 - 38·9)	0·0 (-4·1 - 0·7)	0·0 (-0·4 - 0·8)
*Deaths*	7·6 (6·9 - 8·0)	1·5 (1·4 - 1·6)	9·8 (8·9 - 11·1)	1·3 (1·1 - 1·4)	28·9 (23·2 - 37·4)	0·1 (0·01 - 0·5)	-19·1 (-22·1 - -14·6)	0·0 (-0·1 - 0·2)
*DALYs*	1210·6 (905·7 - 1624·7)	217·8 (154·8 - 302·1)	1375·1 (997·1 - 1861·7)	175·9 (119·4 - 253·3)	13·6 (10·1 - 14·6)	5·5 (3·0 - 7·9)	-19·3 (-22·9 - -16·1)	-1·4 (-2·2 - -1·1)
*YLLs*	443·3 (393·3 - 471·9)	77·8 (70·1 - 81·9)	458·3 (404·9 - 522·5)	58·0 (51·9 - 64·9)	3·4 (3·0 - 10·7)	0·5 (0·2 - 1·7)	-25·5 (-28·0 - -20·6)	-0·7 (-1·6 - -0·3)
*YLDs*	767·2 (460·9 - 1173·5)	140·0 (76·4 - 224·1)	916·8 (537·9 - 1406·3)	117·9 (61·6 - 195·5)	19·5 (16·7 - 21·8)	5·0 (2·6 - 7·8)	-15·8 (-19·3 - -12·8)	-0·7 (-2·5 - -0·1)
High-Income Super Region								
Southern Latin America								
Argentina								
*Prevalence*	76·9 (19·3 - 127·2)	232·2 (58·3 - 384·5)	120·2 (37·4 - 192·9)	268·0 (83·7 - 428·9)	56·4 (51·7 - 93·7)	1·4 (0·6 - 2·2)	15·4 (11·6 - 43·5)	1·2 (0·8 - 1·5)
*Deaths*	251·9 (228·0 - 265·5)	0·8 (0·7 - 0·8)	344·5 (315·8 - 366·3)	0·7 (0·7 - 0·8)	36·7 (35·1- 38·5)	0·0 (0·0 - 0·6)	-7·7 (-9·0 - -6·1)	0·0 (-0·3 - 0·1)
*DALYs*	37·7 (18·7 - 64·3)	114·3 (56·7 - 194·9)	49·7 (25·7 - 88·5)	110·9 (56·9 - 197·7)	31·6 (28·8 - 37·6)	0·4 (0·2 - 0·8)	-3·0 (-5·3 - 0·5)	-0·1 (-0·9 - 0·1)
*YLLs*	13·1 (11·5 - 13·9)	40·1 (35·1 - 42·6)	16·1 (14·5 - 17·2)	35·6 (31·9 - 38·3)	22·8 (20·5 – 24.0)	0·1 (0·1 - 0·4)	-11·1 (-18·9 - -8·0)	-0·1 (-0·5 - 0·2)
*YLDs*	24·6 (05·7 - 50·7)	74·2 (17·3 - 153·3)	33·6 (09·5 - 71·9)	75·2 (21·3 - 161·6)	36·3 (26·3 – 42.0)	0·3 (0·1 - 0·7)	1·3 (0·4 - 9·7)	0·0 (-0·1 - 0·3)
Chile								
*Prevalence*	60·4 (17·6 - 103·0)	449·4 (131·5 - 763·3)	85·2 (24·5 - 134·2)	467·2 (133·9 - 737·6)	41 (39·3 - 46·3)	0·8 (0·2 - 1·0)	4·0 (1·8 - 8·4)	0·6 (0·1 - 0·9)
*Deaths*	214·8 (202·2 - 226·9)	1·7 (1·6 - 1·8)	287·9 (262·9 - 311·7)	1·4 (1·2 - 1·5)	34 (30 - 37·3)	0·0 (0·0 - 0·6)	-21·3 (-33·8 - -19·0)	0·0 (-0·2 - 0·3)
*DALYs*	30·2 (15·7 - 51·9)	223·7 (116·9 - 383·6)	32·5 (15·5 - 56·5)	176·5 (81·6 - 309·9)	7·7 (-1·4 – 9.0)	0·1 (0·0 - 0·2)	-21·1 (-30·2 - -19·2)	-1·6 (-3·2 - -0·5)
*YLLs*	10·9 (10·2 - 11·5)	80·8 (75·9 - 85·3)	11·0 (10·1 - 12·1)	57·2 (52·1 - 63·7)	1·2 (-1·2 - 4·8)	0·0 (0·0 - 0·8)	-29·3 (-31·4 - -25·3)	-0·8 (-1·2 - -0·3)
*YLDs*	19·3 (04·9 - 40·9)	142·9 (36·5 - 302·9)	21·5 (04·5 - 45·5)	119·3 (24·8 - 253·2)	11·4 (-3 - 13·2)	0·1 (0·0 - 0·2)	-16·5 (-32·1 - -11·4)	-0·8 (-1·7 - -0·4)
Uruguay								
*Prevalence*	10·1 (2·8 - 16·9)	321·5 (88·1 - 540·5)	13·4 (3·8 - 21·4)	381·1 (106·9 - 612·2)	32·3 (26·3 - 35·3)	0·1 (0·0 - 0·1)	18·5 (13·3 - 21·4)	2·0 (0·6 - 2·4)
*Deaths*	41·9 (39·4 - 45·1)	1·3 (1·2 - 1·4)	61·3 (52·5 - 66·7)	1·5 (1·3 - 1·6)	46·5 (33·1 - 48·0)	0·0 (0·0 - 0·3)	15·1 (6·7 - 26·2)	0·0 (0·0 - 0·3)
*DALYs*	5·0 (2·6 - 8·8)	161·2 (82·5 - 282·2)	5·8 (2·9 - 10·1)	167·7 (84·9 - 291·9)	16·1 (15·7- 16·5)	0·0 (0·0 - 0·6)	4·1 (2·8 - 8·4)	0·2 (0·1 - 0·3)
*YLLs*	1·9 (1·7 - 2·0)	59·9 (56·0 - 65·6)	2·2 (1·9 - 2·4)	63·3 (56·7 - 70·4)	19·8 (14·6 - 20·3)	0·0 (0·0 - 0·4)	5·7 (1·3 - 7·3)	0·1 (0·0 - 0·2)
*YLDs*	3·2 (0·7 - 6·9)	101·3 (22·4 - 220·6)	3·6 (0·7 - 7·8)	104·5 (20·7 - 226·8)	14·0 (3·4 – 18·5)	0·0 (0·0 - 0·1)	3·1 (-7·6 - 5·8)	0·1 (-0·1 - 0·2)
Latin America and Caribbean Super Region								
Caribbean								
*Prevalence*	134·3 (83·9 - 187·9)	383·5 (238·9 - 534·0)	183·7 (115·1 - 255·5)	386·9 (241·4 - 537·9)	36·8 (36·0 - 37·3)	1·6 (1·0 - 2·3)	0·9 (0·1 - 1·7)	0·1 (-0·1 - 0·1)
*Deaths*	872·7 (629·6 - 1060·9)	2·6 (1·9 - 3·1)	1002·1 (791·2 – 1222.0)	2·1 (1·6 - 2·5)	14·8 (12·2 - 25·7)	0·0 (0·0 - 0·3)	-20·3 (-26·8 - -15·3)	0·0 (0·0 - 0·0)
*DALYs*	96·7 (68·0 - 130·7)	268·8 (191·3 - 361·1)	107·3 (75·8 - 145·5)	228·9 (160·2 - 310·3)	10·9 (8·3 - 11·4)	0·4 (0·3 - 0·5)	-14·9 (-19·3 - -12·1)	-1·3 (-1·7 - -1·0)
*YLLs*	46·6 (24·8 - 74·1)	131·5 (70·8 - 207·7)	57·9 (30·6 - 95·2)	122·6 (64·6 - 202·9)	24·1 (23·1 - 28·5)	0·4 (0·2 - 0·7)	-6·8 (-8·8 - -2·3)	-0·3 (-0·5 - -0·2)
*YLDs*	50·1 (33·0 - 64·2)	137·3 (93·4 - 173·0)	49·4 (36·4 - 62·1)	106·3 (76·9 - 134·0)	-1·3 (-3·3 - 10·3)	0·0 (-0·1 - 0·1)	-22·6 (-27·7 - -12·5)	-1·0 (-1·5 - -0·3)
Andean Latin America								
Bolivia								
*Prevalence*	31·5 (06·9 - 55·6)	509·4 (111·1 - 899·7)	47·1 (11·7 - 79·2)	402·9 (99·7 - 672·9)	49·5 (42·6 - 69·6)	0·5 (0·2 - 0·8)	-20·9 (-25·2 - -15·2)	-3·6 (-7·6 - -0·4)
*Deaths*	197·5 (134·1 - 257·1)	3·4 (2·4 - 4·3)	198·7 (144·6 - 259·6)	1·9 (1·4 - 2·4)	0·6 (-7·8 - 1·0)	0·0 (0·0 - 0·3)	-45·9 (-53·9 - -34·0)	-0·1 (-0·2 - 0·0)
*DALYs*	25·4 (13·7 - 39·7)	387·1 (202·1 - 611·2)	26·0 (12·9 - 43·5)	219·9 (108·0 - 366·4)	2·4 (-5·4 - 9·5)	0·0 (0·0 - 0·1)	-43·2 (-46·5 - -40·0)	-5·6 (-8·2 - -3·1)
*YLLs*	12·2 (07·8 - 16·4)	176·3 (120·5 - 228·2)	09·8 (06·9 - 12·9)	82·3 (58·9 - 108·7)	-19·5 (-21·7 - -11·3)	-0·1 (-0·3 - 0·0)	-53·3 (-61·1 - -42·4)	-3·1 (-4·0 - -2·1)
*YLDs*	13·3 (02·7 - 27·0)	210·9 (42·9 - 429·3)	16·2 (03·7 - 33·7)	137·7 (30·7 - 285·9)	22·5 (16·7 - 24·8)	0·1 (0·0 - 0·2)	-34·7 (-48·5 - -23·4)	-2·4 (-4·8 - -0·4)
Ecuador								
*Prevalence*	60·6 (14·6 - 104·9)	646·9 (156·5 - 1113·2)	114·9 (30·8 - 190·3)	659·7 (177·4 - 1089·2)	89·7 (81·4 - 110·3)	1·8 (0·5 - 2·8)	2·0 (-2·2 - 13·3)	0·4 (0·2 - 0·8)
*Deaths*	298·6 (247·3 - 324·9)	3·3 (2·8 - 3·6)	333·7 (257·8 - 468·3)	1·9 (1·5 - 2·7)	11·8 (4·3 - 44·1)	0·0 (0·0 - 0·12)	-40·7 (-45·4 - -23·7)	0·0 (-0·1 - 0·1)
*DALYs*	40·9 (22·4 - 65·4)	414·4 (220·4 - 668·9)	51·6 (25·1 - 87·5)	293·2 (142·1 - 496·3)	26·4 (12·1 - 33·8)	0·4 (0·1 - 0·7)	-29·2 (-35·5 - -15·8)	-4·0 (-5·8 - -2·6)
*YLLs*	17·6 (14·5 - 19·3)	169·3 (140·5 - 184·3)	16·5 (12·7 - 23·9)	92·4 (71·1 - 133·3)	-6·0 (-12·3 - 24·2)	0·0 (-0·1 - 0·2)	-45·4 (-49·4 - -27·7)	-2·6 (-3·3 - -1·7)
*YLDs*	23·3 (05·2 - 47·6)	245·1 (55·4 - 498·6)	35·2 (8·8 - 69·4)	200·8 (49·9 - 395·7)	50·9 (45·9 - 69·8)	0·4 (0·1 - 0·7)	-18·1 (-29·8 - -10·6)	-1·5 (-3·4 - -0·4)
Peru								
*Prevalence*	97·7 (24·5 - 168·8)	459·7 (119·2 - 786·3)	153·3 (39·3 - 256·5)	452·9 (116·6 - 754·8)	56·9 (52·0 - 60·3)	1·9 (0·5 - 2·9)	-1·5 (-7·2 - -0·4)	-0·2 (-0·5 - 0·0)
*Deaths*	381·2 (285·4 - 441·8)	1·9 (1·4 - 2·2)	288·2 (205·7 - 392·5)	0·8 (0·6 - 1·1)	-24·4 (-27·9 - -11·2)	0·0 (0·0 - 0·21)	-56·3 (-68·3 - -38·9)	0·0 (-0·1 - 0·1)
*DALYs*	59·7 (31·5 - 96·6)	271·6 (141·4 - 440·3)	59·5 (24·6 - 112·2)	174·6 (71·8 - 328·1)	-0·3 (-21·9 - 16·1)	0·0 (-0·2 - 0·5)	-35·7 (-49·2 - -25·5)	-3·2 (-3·7 - -2·3)
*YLLs*	22·7 (17·1 - 26·3)	99·9 (74·5 - 115·9)	14·1 (09·9 - 19·5)	40·5 (28·6 - 56·1)	-38·0 (-42·1 - -25·9)	-0·3 (-0·5 - -0·0)	-59·4 (-61·6 - -51·6)	-2·0 (-2·5 - -1·5)
*YLDs*	37·0 (09·0 - 72·6)	171·8 (41·5 - 336·7)	45·4 (10·6 - 96·3)	134·1 (31·5 - 283·6)	22·8 (17·9 - 32·7)	0·3 (0·1 - 0·8)	-21·9 (-34·2 - -15·8)	-1·3 (-1·8 - -0·3)
Central Latin America								
Colombia								
*Prevalence*	173·9 (42·4 - 314·4)	530·9 (130·2 - 951·1)	258·2 (70·1 - 431·4)	537·1 (146·2 - 899·6)	48·4 (37·2 - 65·3)	2·8 (0·9 - 3·9)	1·2 (-5·4 - 12·3)	0·2 (-1·5 - 0·7)
*Deaths*	529·9 (453·9 - 561·6)	1·7 (1·5 - 1·8)	647·7 (497·7 - 835·1)	1·3 (1·0 - 1·7)	22·2 (9·6 - 48·7)	0·0 (0·0 - 0·1)	-24·9 (-34·0 - -7·7)	0·0 (-0·1 - 0·1)
*DALYs*	92·8 (44·9 - 166·4)	275·3 (131·6 - 500·3)	102·7 (46·6 - 186·5)	214·5 (96·8 - 390·1)	10·7 (3·8 - 12·1)	0·3 (0·1 - 0·7)	-22·1 (-36·5 - -18·0)	-2·0 (-3·7 - -1·2)
*YLLs*	31·6 (26·3 - 33·9)	91·2 (77·5 - 97·1)	31·4 (24·1 - 40·8)	65·6 (50·4 - 85·3)	-0·5 (-8·3 - 20·4)	0·0 (-0·1 - 0·2)	-28·1 (-34·9 - -12·2)	-0·9 (-1·9 - -0·4)
*YLDs*	61·2 (13·9 - 134·6)	184·1 (41·4 - 406·5)	71·3 (15·9 - 156·4)	148·9 (32·8 - 327·2)	16·5 (15·0 - 26·2)	0·3 (0·1 - 0·7)	-19·1 (-22·8 - -12·5)	-1·2 (-2·6 - -0·3)
Costa Rica								
*Prevalence*	14·3 (3·3 - 25·1)	481·6 (110·7 - 838·2)	25·8 (07·5 - 41·2)	543·74 (159·9 - 874·2)	80·6 (64·0 - 130·7)	0·4 (0·1 - 0·5)	12·9 (4·3 - 44·4)	2·1 (1·6 - 3·2)
*Deaths*	39·7 (36·7 - 42·3)	1·6 (1·5 - 1·7)	61·7 (46·6 - 78·8)	1·2 (0·9 - 1·5)	55·2 (27·1 - 86·2)	0·0 (0·0 - 0·09)	-25·8 (-37·4 - -11·5)	0·0 (-0·1 - 0·1)
*DALYs*	6·4 (2·9 - 11·4)	214·6 (99·1 - 385·7)	9·6 (4·0 - 17·4)	199·5 (82·1 - 364·2)	50·6 (40·5 - 52·3)	0·1 (0·0 - 0·2)	-7·1 (-17·1 - -5·6)	-0·5 (-0·7 - -0·2)
*YLLs*	1·9 (1·9 - 2·1)	69·2 (64·5 - 74·0)	2·6 (1·9 - 3·4)	51·6 (39·0 - 67·1)	30·3 (5·1 - 57·0)	0·0 (0·0 - 0·1)	-25·4 (-39·5 - -9·3)	-0·6 (-0·8 - -0·2)
*YLDs*	4·4 (0·9 - 9·5)	145·4 (29·8 - 316·1)	6·9 (1·6 - 14·8)	147·9 (33·9 - 310·4)	59·7 (56·4 - 79·4)	0·1 (0·0 - 0·2)	1·7 (-1·8 - 13·8)	0·1 (-0·1 - 0·2)
El Salvador								
*Prevalence*	20·2 (4·9 - 38·0)	381·9 (91·1 - 709·2)	28·4 (7·2 - 48·3)	449·9 (112·7 - 762·4)	40·2 (27·0 - 44·2)	0·3 (0·1 - 0·6)	17·8 (7·5 - 23·8)	2·3 (0·7 - 2·8)
*Deaths*	74·3 (63·1 - 82·4)	1·6 (1·4 - 1·7)	69·6 (51·6 - 91·7)	1·1 (0·8 - 1·5)	-6·4 (-18·3 - 11·3)	0·0 (0·0 - 0·1)	-27·7 (-39·4 - -12·9)	0·0 (-0·1 - 0·1)
*DALYs*	11·9 (6·0 - 21·1)	219·4 (110·0 - 382·7)	11·8 (4·9 - 21·7)	186·1 (78·6 - 342·0)	-1·7 (-17·9 - 2·5)	0·0 (0·0 - 0·1)	-15·2 (-28·5 - -10·6)	-1·1 (-3·0 - -0·4)
*YLLs*	4·5 (3·6 - 5·1)	80·8 (67·8 - 89·7)	03·1 (2·3 - 4·1)	49·6 (36·8 - 65·7)	-30·6 (-35·6 - -18·4)	0·0 (0·0 - 0·0)	-38·5 (-45·7 - -26·8)	-1·0 (-2·0 - -0·8)
*YLDs*	7·5 (1·7 - 16·3)	138·6 (30·8 - 300·1)	08·6 (1·8 - 18·2)	136·5 (28·9 - 288·9)	15·7 (9·1 - 22·0)	0·0 (0·0 - 0·1)	-1·6 (-6·4 - -0·7)	-0·1 (-0·8 – 0·0)
Guatemala								
*Prevalence*	34·8 (07·4 - 66·7)	453·3 (97·7 - 871·4)	84·6 (22·1 - 147·5)	479·9 (126·5 - 828·3)	143·3 (127·5 - 151·1)	1·7 (0·5 - 2·7)	5·9 (-4·9 - 29·5)	0·9 (0·5 - 1·4)
*Deaths*	254·0 (215·0 - 285·5)	3·7 (3·2 - 4·2)	443·1 (346·3 - 565·5)	2·7 (2·2 - 3·5)	74·4 (61·1 - 98·1)	0·0 (0·0 - 0·1)	-26·2 (-33·1 - -15·9)	0·0 (-0·1 - 0·1)
*DALYs*	29·8 (18·6 - 46·4)	372·9 (229·4 - 578·5)	52·8 (29·9 - 83·4)	292·5 (164·5 - 465·7)	77·1 (60·6 - 79·6)	0·8 (0·4 - 1·2)	-21·6 (-28·3 - -15·5)	-2·7 (-3·8 - -1·2)
*YLLs*	15·6 (12·9 - 17·6)	191·1 (161·9 - 214·9)	24·4 (19·0 - 31·5)	133·8 (104·5 - 171·3)	56·6 (46·7 - 78·9)	0·3 (0·2 - 0·5)	-30·0 (-35·5 - -20·3)	-1·9 (-2·9 - -1·5)
*YLDs*	14·2 (02·8 - 30·7)	181·8 (35·6 - 388·5)	28·3 (5·9 - 58·7)	158·7 (33·4 - 324·9)	99·7 (91·5 - 115·5)	0·5 (0·1 - 0·9)	-12·7 (-16·4 - -6·3)	-0·8 (-2·1 - -0·1)
Honduras								
*Prevalence*	24·5 (5·0 - 46·0)	536·5 (109·7 - 995·6)	49·3 (13·4 - 84·5)	514·8 (139·6 - 869·6)	101·4 (83·6 - 167·1)	0·8 (0·3 - 1·3)	-4·0 (-12·6 - 27·2)	-0·7 (-4·2 - 0·2)
*Deaths*	164·9 (126·9 - 191·4)	4·2 (3·4 - 4·9)	239·3 (165·9 - 332·5)	3·2 (2·3 - 4·4)	45·1 (30·7 - 73·7)	0·0 (0·0 - 0·1)	-22·9 (-32·8 - -11·0)	0·0 (-0·1 - 0·1)
*DALYs*	19·7 (11·7 - 30·2)	415·9 (245·8 - 635·4)	27·2 (13·6 - 45·3)	288·9 (147·2 - 472·9)	38·2 (15·9 - 50·0)	0·3 (0·1 - 0·5)	-30·5 (-40·1 - -25·6)	-4·2 (-5·4 - -3·3)
*YLLs*	10·1 (7·5 - 11·9)	209·1 (160·8 - 243·2)	10·7 (7·1 - 15·3)	118·7 (79·9 - 167·6)	6·2 (-5·7 - 28·7)	0·0 (0·0 - 0·1)	-43·2 (-50·3 - -31·1)	-3·0 (-3·7 - -2·5)
*YLDs*	9·6 (1·9 - 19·8)	206·8 (40·9 - 423·9)	16·5 (3·8 - 34·2)	170·2 (39·4 - 351·7)	71·8 (63·1 - 99·4)	0·2 (0·1 - 0·5)	-17·7 (-27·0 - -3·6)	-1·2 (-2·4 - 0·0)
Mexico								
*Prevalence*	552·5 (391·2 - 746·8)	661·9 (472·4 - 888·2)	778·4 (549·8 - 1020·2)	627·2 (443·9 - 820·6)	40·9 (36·6 - 46·6)	7·5 (5·3 - 9·1)	-5·3 (-7·6 - -2·0)	-1·2 (-2·3 - -0·9)
*Deaths*	1834·5 (1604·5 - 1933·6)	2·5 (2·2 - 2·6)	2166·3 (1857·7 - 2563·3)	1·8 (1·5 - 2·1)	18·1 (15·8 - 32·6)	0·0 (-0·1 - 0·0)	-29·4 (-32·6 - -19·8)	0·0 (-0·1 - 0·1)
*DALYs*	307·5 (226·9 - 419·8)	356·5 (263·8 - 476·5)	331·9 (238·1 - 450·0)	266·3 (190·9 - 360·1)	7·9 (5·0 - 9·2)	0·8 (0·4 - 1·0)	-25·3 (-30·6 - -20·4)	-3·0 (-3·9 - -2·4)
*YLLs*	108·6 (92·0 - 116·5)	122·9 (107·2 - 130·1)	102·0 (88·4 - 119·7)	81·4 (70·5 - 94·8)	-6·0 (-9·0 - 2·8)	-0·2 (-0·3 - 0·0)	-33·8 (-39·2 - -27·1)	-1·4 (-3·2 - -1·2)
*YLDs*	198·9 (118·2 - 309·3)	233·5 (141·1 - 354·9)	229·9 (133·9 - 344·3)	184·9 (108·1 - 276·5)	15·5 (13·3 - 21·3)	1·0 (0·5 - 1·2)	-20·8 (-33·4 - -18·1)	-1·6 (-3·1 - -0·6)
Nicaragua								
*Prevalence*	16·2 (3·9 - 31·2)	415·4 (101·4 - 782·4)	26·5 (06·4 - 45·7)	408·2 (97·3 - 695·8)	63·3 (46·5 - 72·6)	0·3 (0·1 - 0·5)	-1·7 (-11·1 - -4·0)	-0·2 (-2·0 - 0·1)
*Deaths*	76·6 (57·8 - 89·1)	2·3 (1·8 - 2·5)	77·7 (62·3 - 99·6)	1·4 (1·2 - 1·8)	1·3 (0·8 - 8·8)	0·0 (-0·1 - 0·3)	-36·4 (-47·3 - -28·0)	0·0 (-0·1 - 0·1)
*DALYs*	10·9 (5·9 - 17·9)	262·4 (141·7 - 432·8)	11·6 (5·5 - 20·8)	178·7 (84·9 - 320·8)	5·8 (-8·4 - 16·0)	0·0 (-0·2 - 0·1)	-31·9 (-40·1 - -25·9)	-2·8 (-3·7 - -1·9)
*YLLs*	5·0 (3·6 - 6·1)	114·0 (87·9 - 130·5)	3·7 (2·9 - 04·9)	58·7 (46·7 - 75·9)	-26·1 (-37·8 - -19·8)	0·0 (0·0 - 0·1)	-48·5 (-57·0 - -41·8)	-1·8 (-2·4 - -1·0)
*YLDs*	5·9 (1·3 - 12·9)	148·3 (31·2 - 318·2)	7·9 (1·7 - 16·9)	119·9 (26·1 - 258·7)	33·0 (31·4 - 34·7)	0·1 (0·0 - 0·3)	-19·1 (-26·1 - -18·7)	-0·9 (-2·0 - -0·2)
Panama								
*Prevalence*	9·8 (2·5 - 17·3)	415·4 (103·5 - 726·9)	22·6 (6·5 - 36·3)	543·2 (157·1 - 871·7)	131·7 (109·5 - 167·2)	0·4 (0·1 - 0·6)	30·8 (19·9 - 51·8)	4·3 (1·8 - 4·8)
*Deaths*	28·9 (26·7 - 32·4)	1·4 (1·3 - 1·6)	62·9 (48·2 - 81·2)	1·5 (1·2 - 1·9)	117·3 (80·4 - 151·0)	0·0 (-0·2 - 0·2)	4·9 (-13·5 - 24·4)	0·0 (-0·1 - 0·1)
*DALYs*	4·7 (2·2 - 8·2)	195·9 (92·9 - 342·8)	9·0 (04·3 - 15·9)	215·8 (101·9 - 383·6)	93·7 (83·4 - 104·9)	0·1 (-0·1 - 0·3)	10·2 (9·6 - 11·9)	0·7 (0·3 - 1·4)
*YLLs*	1·5 (1·3 - 1·7)	62·2 (57·2 - 70·9)	2·7 (02·0 - 3·5)	64·6 (48·6 - 84·1)	84·5 (53·1 - 106·6)	0·0 (-0·1 - 0·1)	3·8 (-14·9 - 18·5)	0·1 (-0·3 - 0·4)
*YLDs*	3·2 (0·8 - 6·7)	133·7 (31·5 - 280·2)	6·3 (01·6 - 13·2)	151·3 (37·9 - 317·2)	98·0 (95·7 - 110·3)	0·1 (0·0 - 0·2)	13·1 (8·4 - 18·2)	0·6 (0·2 - 1·2)
Venezuela								
*Prevalence*	107·5 (27·7 - 189·5)	591·7 (151·1 - 1038·5)	156·4 (48·5 - 261·9)	558·2 (172·4 - 936·9)	45·5 (38·2 - 75·0)	1·6 (0·7 - 2·4)	-5·6 (-9·8 - 5·1)	-1·1 (-3·4 - 0·7)
*Deaths*	350·0 (311·6 - 368·2)	2·2 (1·9 - 2·3)	527·2 (389·8 - 687·6)	1·8 (1·3 - 2·3)	50·6 (25·1 - 86·7)	0·0 (0·0 - 0·1)	-19·3 (-32·3 - 0·0)	0·0 (-0·1 - 0·1)
*DALYs*	56·4 (28·1 - 95·9)	306·4 (154·0 - 521·9)	68·8 (33·3 - 120·8)	243·3 (116·8 - 430·9)	22·1 (18·5 - 26·0)	0·4 (0·2 - 0·8)	-20·6 (-24·2 - -17·4)	-2·1 (-3·0 - -1·2)
*YLLs*	18·9 (16·9 - 19·9)	103·5 (92·2 - 108·8)	23·4 (17·2 - 30·5)	80·7 (59·6 - 105·1)	23·6 (1·7 - 53·0)	0·1 (0·0 - 0·4)	-22·0 (-35·3 - -3·4)	-0·8 (-1·1 - -0·1)
*YLDs*	37·5 (09·0 - 76·7)	202·9 (50·3 - 415·8)	45·4 (11·5 - 96·9)	162·5 (41·2 - 349·2)	21·3 (17·6 - 26·4)	0·3 (0·1 - 0·7)	-19·9 (-28·0 - -16·0)	-1·3 (-2·2 - -0·3)
Tropical Latin America								
Brazil								
*Prevalence*	719·2 (468·9 - 993·7)	500·8 (325·9 - 688·9)	940·3 (641·2 - 1245·9)	429·9 (291·5 - 572·0)	30·7 (25·4 - 36·7)	7·4 (5·7 - 8·4)	-14·2 (-17·6 - -11·0)	-2·4 (-3·9 - -1·1)
*Deaths*	1966·9 (1829·4 - 2082·2)	1·5 (1·4 - 1·5)	2933·9 (2753·4 - 3086·5)	1·3 (1·2 - 1·4)	49·2 (30·5 - 48·2)	0·0 (-0·3 - 0·1)	-11·6 (-22·4 - -7·5)	0·0 (-0·1 - 0·1)
*DALYs*	366·7 (254·3 - 508·2)	248·7 (172·9 - 342·6)	404·2 (287·9 - 549·6)	185·4 (132·9 - 251·9)	10·2 (8·1 - 13·2)	1·2 (1·1 - 1·4)	-25·4 (-33·2 - -16·5)	-2·1 (-3·0 - -1·3)
*YLLs*	114·4 (104·9 - 123·3)	75·9 (70·0 - 81·3)	130·8 (122·4 - 139·6)	59·9 (55·3 - 64·9)	14·4 (13·2 - 16·6)	0·5 (0·1 - 0·8)	-21·1 (-25·0 - -10·2)	-0·5 (-1·5 - -0·2)
*YLDs*	252·3 (143·5 - 392·7)	172·8 (98·9 - 266·3)	273·3 (156·2 - 420·6)	125·5 (71·5 - 193·8)	8·3 (7·1 - 8·8)	0·7 (0·4 - 0·9)	-27·4 (-37·8 - -13·2)	-1·6 (-2·4 - -0·9)
Paraguay								
*Prevalence*	14·8 (3·3 - 26·6)	379·4 (83·1 - 667·9)	28·5 (8·5 - 46·9)	414·9 (124·4 - 683·2)	92·1 (76·4 - 161·1)	1·9 (0·5 - 2·9)	9·3 (2·3 - 29·7)	1·2 (0·5 - 1·4)
*Deaths*	50·3 (44·1 - 57·2)	1·4 (1·2 - 1·6)	87·9 (65·4 - 113·8)	1·3 (0·9 - 1·7)	74·6 (48·2 - 98·9)	0·0 (-0·1 - 0·3)	-7·7 (-21·1 - 4·9)	0·0 (-0·1 - 0·1)
*DALYs*	8·2 (3·9 - 14·1)	205·9 (96·9 - 353·9)	13·2 (6·4 - 22·9)	189·2 (90·7 - 328·1)	60·0 (34·6 - 67·4)	0·0 (-0·2 - 0·5)	-8·1 (-11·4 - -5·3)	-0·6 (-0·9 - -0·2)
*YLLs*	2·9 (2·6 - 3·3)	71·3 (61·9 - 81·0)	04·3 (3·2 - 5·6)	60·6 (44·9 - 78·7)	48·6 (25·3 - 69·9)	-0·3 (-0·5 - -0·1)	-14·9 (-27·4 - -2·8)	-0·4 (-0·6 - -0·1)
*YLDs*	5·3 (1·0 - 11·2)	134·6 (25·9 - 278·7)	08·9 (2·3 - 18·7)	128·6 (33·5 - 268·2)	66·2 (53·5 - 76·8)	0·3 (0·1 - 0·8)	-4·5 (-19·0 - -3·8)	-0·2 (-0·4 - 0·3)

UI: uncertainty interval; DALYs, Disability-adjusted life years; YLLs, years of life lost; YLDs, years lived with disability·

### Risk factor

The only risk quantified in GBD for idiopathic epilepsy was alcohol use, estimated to be responsible for 17·04 % (95% UI 12·6 to 21·8) of the age-standardized DALYs from epilepsy in LAC in 2019. This represents a change of -0·7% from 1990. The associated risk was almost double for men (22·3 %; 95% UI 16·5 to 28·1) compared to women (11·3 %; 95% UI 7·7 to 15·1) in 2019. Besides, a higher percentage was found in people from the 15 to 69 years old cluster (23·8 %; 95% UI 17·6 to 30). Those proportions did not change from 1990. Finally, the countries with higher associated risk were Chile, Argentina, and Paraguay in both 1990 and 2019 (Figure 5).

### Correlation analysis

**Figure 6:**
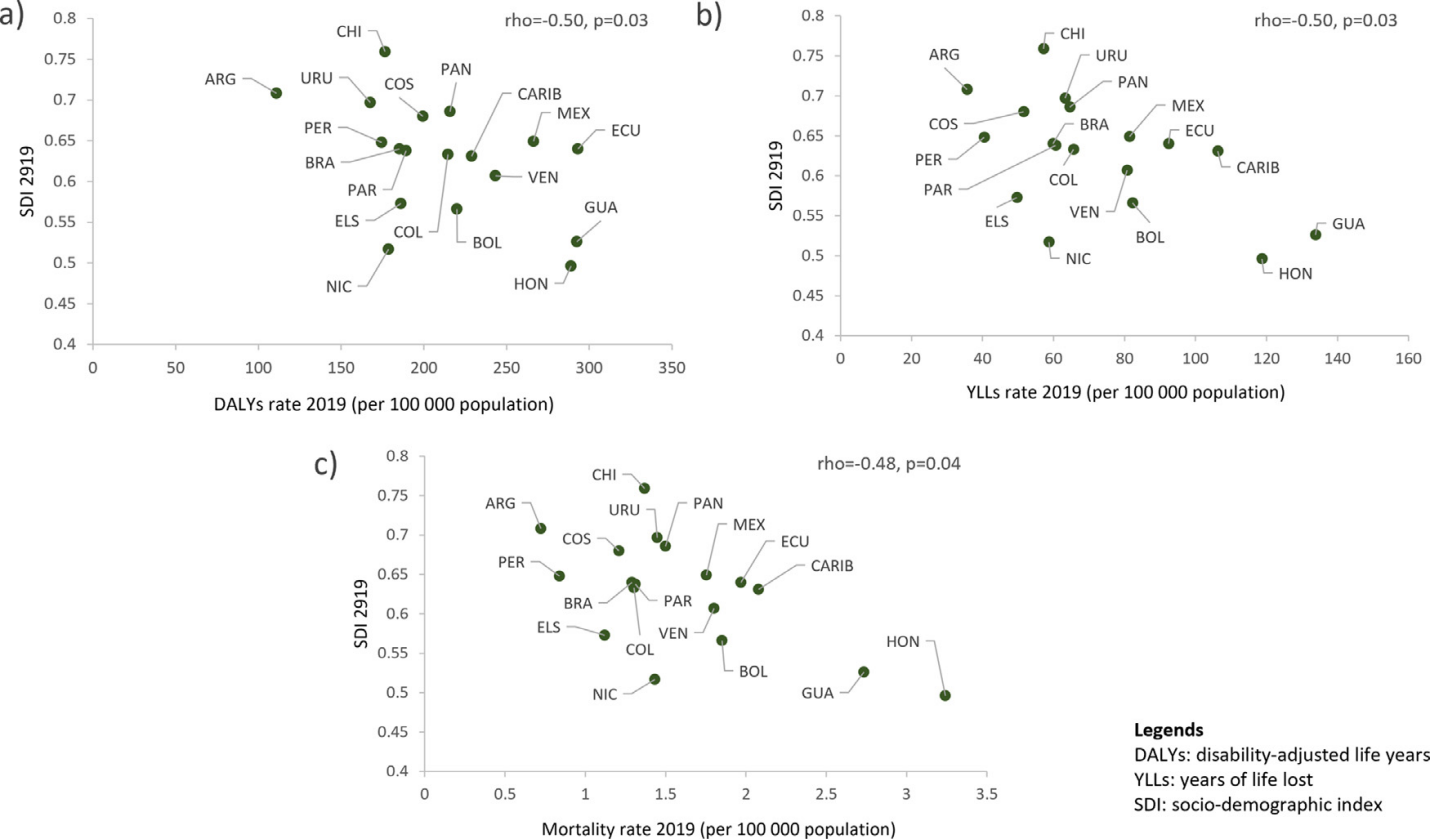
Correlation among burden of disease metrics and SDI in Latin America and the Caribbean in 2019; a) DALYs, b) YLLs, and c) mortality rate.

DALYs, Disability-adjusted life years; YLLs, years of life lost; SDI, socio-demographic index. ARG=Argentina, BOL=Bolivia, BRA=Brazil, CARIB=Caribbean, CHI=Chile, COL=Colombia, COS=Costa Rica, ECU=Ecuador, ELS=El Salvador, GUA=Guatemala, HON=Honduras, MEX=Mexico, NIC=Nicaragua, PAN=Panama, PAR=Paraguay, PER=Peru, URU=Uruguay, VEN=Venezuela.

After the Spearman correlation analysis, we found a negative correlation between the age-standardized rate of death (rho= -0·51; p=0·02), DALYs (rho= -0·55; p=0·01), YLLs (rho= -0·60; p=0·007) and YLDs (rho=-0·47; p=0·04) and the Sociodemographic Index (SDI) in 1990. In 2019, a similar trend was found for the age-standardized rate of death (rho=-0·48, p=0·04), DALYs (rho= -0·50, p= 0·03) and YLL (rho=-0·50; p= 0·03) and SDI (Figure 6), but no for the YLD rate (rho=-0·35, p=0·157). However, we found no statistically significant correlation among the age-standardized prevalence rate with the SDI in 1990 (rho=-0·25; p= 0·33) and in 2019 (rho=-0·01; p= 0·98).

## Discussion

### Main findings

From 1990 to 2019, the burden of epilepsy decreased around 20% in LAC, driven by YLLs reduction; however, the prevalence rate presented no changes for idiopathic epilepsy and an increase for epilepsy with identifiable aetiology. The burden reduction was higher in children and adults, but DALYs remained stable for older adults and even the mortality increased for this age group. For 2019, we found that 6·34 million people live with active epilepsy in LAC (3·11 million with idiopathic epilepsy and 3·22 million with epilepsy with identifiable aetiology), representing 8·1% of the total people with this diagnosis worldwide. Likewise, LAC countries concentrated 9·5% of the total global DALYs for epilepsy, ranking three among the GBD regions (after Central Asia and Sub-Saharan Africa regions).^2^ The countries with the highest burden were Ecuador and Guatemala, and those with the lowest estimates were Argentina and Uruguay. The current epilepsy burden in LAC is disability predominant (YLDs rates), mostly in men compared to women, and in the youth and elderly. However, the death-related estimates are still elevated than in other GBD regions,^2^ of which are higher in male older adults. Alcohol use is the only available risk factor for idiopathic epilepsy in LAC, which is associated with 17% of the reported DALYs. The SDI is an important regional determinant of this burden (higher SDI, less epilepsy burden, and mortality) but not of the frequency and associated disability of the disease.

### Epilepsy prevalence in LAC

We found that the 2019 prevalence rate of all-causes epilepsy in LAC was 8·17 per 1,000 inhabitants and 4·12 per 1,000 inhabitants for idiopathic epilepsy. According to the most recent meta-analysis, the prevalence of active epilepsy of all causes in LAC was 9·06 per 1,000 inhabitants,^3^ which is very close to the GBD study estimates. Besides, it is known that around 40% of the epilepsies are of idiopathic aetiology,^19^ hence our results are within what is expected. Also, there was no available data to calculate the prevalence of epilepsy cases by each identifiable aetiology. Previous systematic reviews did not provide a prevalence estimate by the aetiology,^3^^,^^20^ since most primary studies were published prior to the publication of the 2017 ILAE statement.^21^ However, it is known that around 17% of the patients living with epilepsy in LAC have neurocysticercosis (NCC) as the aetiology.^3^ NCC related epilepsies do not have an independent entry in the GBD studies, neither as aetiology nor as a risk factor. Hence, there is a need for improving the real estimate to all causes-active epilepsy burden in the GBD study. Finally, our results show the prevalence rate of epilepsy had not changed from 1990 to 2019, which had also been described previously after cumulative meta-analysis, ^3^ meaning that the prevalence estimate is s, thus there are as many new cases as deceased.

### Insights from country-level data

Ecuador had the highest prevalence rate and had the highest burden (DALYs) with a YLD predominance. This means that Ecuador has the greatest burden of epilepsy-related disability. Ecuador belongs to the WHO epidemiological region D, due to its high overall children and adult mortality rate.^22^ Around 37% of its population lives in rural settings,^23^ which is associated with a higher treatment gap in epilepsy.^20^

Since there is limited access to neuroimaging or immuno-assays, many secondary epilepsies are considered idiopathic, including those due to NCC. Still, there is important underreporting of those diagnosed with NCC-related epilepsy, mostly from the private health system.^24^ Patients with epilepsy in Ecuador had odds to have NCC three times higher than those without epilepsy.^25^ Thus, NCC could explain partially the high burden of epilepsy in Ecuador due to case aetiology underdiagnosis.

Guatemala and Honduras also had a high rate of DALYs, but with YLL predominance and the highest mortality rates. These countries are known for the health inequities^26^ presenting the lowest scores of HDI in the region,^27^ and high rates of hunger and malnutrition.^28^ Like Ecuador, NCC is prevalent in these tropical countries,^29^ and contributes as an undiagnosed aetiology to the burden.

Besides, the lack of access to proper and timely epilepsy management (pharmacological or surgical) in these countries,^30^^,^^31^ could explain the high mortality since improved seizure control by treatment is known to be the most important measure to prevent SUDEP and accidents. ^32^^,^^33^ Other causes of early death in people living with epilepsy in Latin America reported were status epilepticus, brain tumours, stroke, suicide, pneumonia, and sepsis. ^30^

Finally, the stigma for people with epilepsy in LAC remains an issue,^34^ which could impair the willingness to seek treatment and rehabilitation and increase the consequent mortality and disability.

Argentina and Uruguay had the lowest prevalence rate and the lowest burden of epilepsy in terms of DALYs and both YLD and YLL rates. The prevalence in older adults is smaller than the other age groups, one possible explanation is that both countries have universal insurance coverage, the highest income of the region, and a better sanitation system.^35^ We argue that some conditions intrinsic to this area could be explaining these great differences, like the southern location far from tropical areas (then less risk of NCC), ^10^ and perhaps the smaller rate of endogamy. ^36^ Also, we found that a great proportion of DALYs were explained by alcohol use. This opens the possibility for public health policies to control this risk factor and lower the burden even more.

### The role of the social development index

Prevalence was not correlated with SDI, suggesting that the index increase itself cannot change the natural history of idiopathic epilepsies. However, the number of included estimates was small and could be masking a potential correlation. A positive association between socioeconomic determinants of health and epilepsy were reported in previous population-based epidemiological studies in LAC.^3^

We found that DALYs and YLLs in 1990 and 2019 were lower in countries with higher SDI according to our correlation analysis, corroborating the global results.^2^^,^^37^ Economic development contributes toreducing the burden due to mortality reduction, through earlier access to diagnosis and treatment, higher educational level, and reduction of secondary causes associated with poor sanitation (such as NCC).^3^ Countries with lower SDI present higher mortality since a substantial treatment gap is still present due to lack of health care resources and treatment availability.^2^^,^^37^^,^^38^

However, YLDs was lower in countries with higher SDI in 1990 but not in 2019. These results suggest that the economic development may only improve epilepsy-related disability in a limited way since other factors such as an organized healthcare system, strong primary care, determinants of medication adherence, successful self-management, stigma, and work opportunities are lacking due to political, social, and cultural aspects, which are independent of only economic growth.^39^, ^40^, ^41^

### Sex differences

Prevalence and YLDs were not different among men and women. However, DALYs, YLLs, and mortality are higher in males. These results are aligned with global findings.^2^ Some hypotheses could explain the higher premature death of men with epilepsy in LAC. First, it has been shown a higher susceptibility to hyperexcitability events and occurrence of epileptic seizures in men than women. Changes in seizure sensitivity could be attributed to steroid hormones,^42^ including fluctuations in neurosteroids as well as neuroplasticity in their receptor signalling systems; however, the molecular mechanisms remain unclear.^43^ Second, men with epilepsy have more exposure to risk factors and potential causes of injury,^44^ such as alcohol use (as we found in our risk factor analysis),^45^ labour and traffic accidents,^46^ and suicide.^47^ Finally, some studies found a higher incidence of SUDEP in males,^48^, ^49^, ^50^ which may also be associated with alcohol consumption.^48^ These factors could explain the higher burden and mortality of men in LAC.

### Age-group differences

In 2019, the burden was higher in the 70+ age group, while it decreased by 27% for the under 5 years group and remain still in other group ages. The age-related burden curve in LAC has three main characteristics: a) shift to the right (the peaks are later), b) higher estimates in the curve's valley (especially high YLLS in adults), c) higher estimates (YLDs and YLLs) in older adults – the second peak is higher than the first peak (especially YLDs).

Countries in Latin America had increased their national income in the past 30 years and improved their insurance coverage.^51^ This translated into a lower prevalence of neonatal encephalopathy and hypoxic-ischemic encephalopathy,^52^ better maternal-neonatal care,^53^ and relative improvement of CNS infections.^54^ Those factors could explain the burden reduction in children.

On the other hand, we found no changes for the 70+ group with the highest mortality rate and highest burden for disability.

Since our estimate includes mainly idiopathic epilepsy, the burden in older adults would be underestimated as secondary causes, mostly structural or metabolic are of high prevalence in this age group.^55^ Moreover, idiopathic epilepsies are less likely to remit,^56^ thus it is expected that prevalence and the overall burden due to disability (YLD) would cumulate in older age clusters.

When compared to other age groups, epilepsy in older adults has a higher frequency of acute symptomatic seizures (after an acute brain-altering event), thus higher prevalence of active epilepsy, and a lower frequency of drug-resistant epilepsy, hence a better seizure control with AED monotherapy.^57^ However, there is a lack of access to treatment in LAC, it has been reported that the treatment gap in lower- and upper-middle-income countries remains as high as 70%, especially in rural areas,^58^ which in LAC represents from 10 to 40% of all settings.^23^ Also, the treatment gap could contribute significantly to the mortality estimates in LAC.^20^ Aging in LAC is a fast-pacing, economic, and social challenging situation.^59^ LAC is the region with the largest expected increment in the older adult population (11.6%) worldwide by 2050.^60^ Therefore, the proper diagnosis and timely management of epilepsy in the elderly is an unattended public health priority in this ongoing ageing region.

### Strengths and limitations

This study is the first regional and country-level assessment of the epilepsy burden in LAC and its changes over the past 30 years. We report not only the disease frequency but also the health loss due to epilepsy, allowing us to evaluate the magnitude of epilepsy-related deaths and disability. However, some limitations need to be disclosed about the present study. First, this is a secondary data analysis, so we relied upon the exhaustivity and robustness of a previous research effort, important limitations of the GBD data include the definition of epilepsy with identifiable aetiology and the description of epilepsy-related deaths Second, the presence of simulated values for countries with sparse data could affect the precision of our estimates. Third, there is no availability of YLLs and mortality data on epilepsy with identifiable aetiology, the description of most common etiologies, and data on important risk factors such as sanitation or cysticercosis. Third, some of the points estimates differences found in our results represented only a trend since they had overlapping confidence intervals, such as age-based differences in prevalence rates, regional differences in DALYs and YLDs rates and country-based differences in YLLs and YLDs. Finally, there is a risk of overestimating the idiopathic epilepsy burden due to diagnostic uncertainty (EEG and neuroimaging availability) in the region, especially associated with neuro infections (unrecognized neurocysticercosis).

### Implications for public health and research

Based on our findings, we encourage urgent public health actions in LAC. First, since there is a lack of neurologists especially in the rural areas, most of the epilepsy care falls on the primary care physicians, who do not always have the necessary skills. Such a problem could be addressed throughout a systematic primary care physician (PCP) training on epilepsy diagnosis and management in LAC, with a special focus on late-onset epilepsy. Examples of this strategy are the mental health GAP program^61^ and the Latin American E-learning initiative^62^ which need further implementation in the region. However, to make this solution feasible, online courses should include a learning network that connects epileptologists and PCPs. One example of this approach is the ECHO Ontario program. Second, we suggest the creation of Epilepsy national programs in LAC following the Pan American Health Organization (PAHO) recommendations,^63^ which will help to increase epilepsy awareness and provide guidelines for prevention, management, and organization of all levels of care (First level [primary care including rural areas]: nurses and PCPs; second level [hospitals]: well-trained general neurologist and internists; third level [specialized center]: epileptologists and specialized neurosurgeons), besides, this program should integrate these levels using telemedicine tools. Due to the current COVID-19 pandemic telemedicine reform, this approach seems to be feasible in the region.^64^ Besides the access for specialized physicians, accessing pharmacological and surgical treatments is essential for adequate epilepsy management, therefore, the availability of antiepileptic drugs in all levels of care (selecting “classic” and unexpensive drugs such as valproate or carbamazepine, which have similar efficacy compared to new drugs^65^) has to be prioritized during the ongoing health reform in the region.^66^ Third, we have identified that alcohol use is associated with almost 20% of DALYs in LAC, hence, there is a need for reinforcement of and collaboration with current substance abuse prevention programs to reduce the alcohol consumption in LAC, such as taxation as increased pricing policies as suggested by the PAHO.^67^ The stimulation of more mindful and less use of this substance may lead to a decrease of complications of epilepsy, as SUDEP, and several other alcohol-related health problems.^68^ Finally, the prevention of epilepsy as a public health action should underscore the importance of healthy ageing on the burden of epilepsy in older people. Some risk factors in older people that we could target with public health programs are vascular risk (hypertension, atrial fibrillation, dyslipidemia, and obesity), sleep deprivation, and medication side effects.^55^ Therefore, we need to increase the awareness in general population and physicians of this risk modification and its impact on late-onset epilepsy burden, and to include epilepsy incidence and mortality as metrics of success for healthy aging programs in the region.

## Conclusion

Over the past 30 years, the epilepsy burden in LAC has decreased; however, it is still high and contributes significantly to the global epilepsy burden. This burden is YLD predominant, mostly in the youth and elderly. However, mortality and premature death are still higher than in other GBD regions, particularly in male older adults. Alcohol use is the only available risk factor, and the SDI is an essential regional determinant for burden and mortality (higher SDI, less burden). The high mortality and YLLs in the region, mainly in the elderly, suggest a lack of access to adequate pharmacological treatment. Therefore, there is an urgent need for planning in LAC. This should include prompt access to treatment in all levels of care, underscoring the strengthening of primary care and the systematic reduction of stigma and marginalization of people with epilepsy in LAC.

## Contributors

KPB: Conceptualization, data curation, formal analysis, visualization, writing-original draft, writing - review & editing. ANF: Conceptualization, data curation, visualization, writing-original draft, writing-review & editing. ACR: Conceptualization, data curation, interpretation of the results, writing of the manuscript, and approval of its final version. PSM: Design, interpretation of the results, writing of the manuscript, and approval of its final version. EUK: Design, interpretation of the results, writing of the manuscript, and approval of its final version. CAD: Design, interpretation of the results, writing of the manuscript, and approval of its final version. FF: Design, interpretation of the results, writing of the manuscript, and approval of its final version. JGB: Design, supervision, interpretation of the results, writing of the manuscript, and approval of its final version.

## Data sharing

The data utilize for the displays and calculations presented in this manuscript could be downloaded at http://ghdx.healthdata.org/gbd-results-tool.

**Editor note:***The Lancet* Group takes a neutral position with respect to territorial claims in published maps and institutional affiliations.

## Declaration of interests

The authors declare to have no compelling interests in this article.

## Appendix Supplementary materials

Image, application 1

Image, application 2
