# Supplementary material for: Burden of epilepsy in Latin America and The Caribbean: a trend analysis of the Global Burden of Disease Study 1990 – 2019
Source: Lancet Reg Health Am. 2021 Dec 16;8:100140. doi: 10.1016/j.lana.2021.100140 (PMC9904123; doi:10.1016/j.lana.2021.100140)
Supplement: Supplementary file 1 [file mmc1.docx]

***Editorial disclaimer:*** *This translation in Portuguese was submitted by the authors and we reproduce it as supplied. It has not been peer reviewed. Our editorial processes have only been applied to the original abstract in English, which should serve as reference for this manuscript.*

**RESUMO**

**Contexto:** A prevalência da epilepsia na América Latina e Caribenha (ALC) tem permanecido alta nos últimos 20 anos. Dados sobre o impacto da epilepsia nessas regiões são necessários para o planejamento de e alocação de recursos para políticas de saúde. Porém, não há análises sistemáticas realizadas em relação ao impacto da epilepsia na ALC.

**Métodos:** Nós extraímos dados referentes a todos os países da ALC provenientes do estudo Global Burden of Disease (GBD) realizado entre os anos de 1990 e 2019. O impacto da epilepsia foi mensurado pela prevalência, mortalidade e anos de vida perdidos ajustados por incapacidade - disability-adjusted life-years (DALYs; este é definido pela soma dos anos de vida perdida – years of life lost [YLLs] – para mortalidade prematura e dos anos vividos com incapacidade – years lived with disability [YLDs]), por idade, sexo, ano e país. Números absolutos, taxas e intervalos de incerteza de 95% foram reportados. Nós realizamos uma análise de correlação entre as métricas de impacto e o índex sociodemográfico (ISD).

**Resultados:** O impacto da epilepsia reduziu em torno de 20% na ALC, liderado por uma redução dos YLLs. Em 2019, nós observamos que 6,3 milhões de pessoas estavam vivendo com epilepsia ativa (II 95% 5,3 – 7,4), com 3,22 milhões (II 95% 2,21 – 4,03) e 3,11 milhões (II 2,21 – 4,03) de casos de epilepsia com etiologia identificada e etiologia idiopática, respectivamente. O número de DALYs representou 9,51% (1,37 milhões, II 95% 0,99 – 1,86) do impacto total da epilepsia em 2019. O impacto normalizado pela idade foi 175,88 por 100.000 pessoas (II 95% 119,43 – 253,33), que teve uma distribuição de idade bimodal (maior nos jovens e idosos) direcionada pelas taxas de YLDs. O impacto foi maior em homens e idosos, primeiramente por conta do alto YLLs e alta mortalidade. O uso de álcool foi associado com 17% do DALYs reportado. As estimativas do ISD influenciaram significativamente esse impacto (países com maior ISD apresentaram menos impacto e mortalidade causados pela epilepsia, mas não prevalência ou incapacidade).

**Interpretação:** O impacto da epilepsia tem diminuído na ALC nos últimos 30 anos. Entretanto, a ALC continua ranqueada como a terceira região com o maior impacto global causado pela epilepsia. Essa redução foi maior em crianças, mas impacto e mortalidade cresceram nos idosos. O impacto da epilepsia é predominantemente causador de incapacidade; porém, as estimativas relacionadas a mortalidade continuam mais elevadas do que em outras regiões. O consumo de álcool e o desenvolvimento dos países são determinantes importantes desse impacto. Melhorar o acesso a saúde e o cuidado com a epilepsia na ALC é uma necessidade urgente, particularmente para os idosos. O fortalecimento da rede de atenção primária com ensino a distância e ferramentas da telemedicina, promovendo a modificação de fatores de risco, deve ser priorizada na região.

**Financiamento:** Esse trabalho foi financiado pelos próprios autores.

**Palavras-chave:** Impacto da doença, epilepsia, epidemiologia.
