# Supplementary material for: Burden of epilepsy in Latin America and The Caribbean: a trend analysis of the Global Burden of Disease Study 1990 – 2019
Source: Lancet Reg Health Am. 2021 Dec 16;8:100140. doi: 10.1016/j.lana.2021.100140 (PMC9904123; doi:10.1016/j.lana.2021.100140)
Supplement: Supplementary file 2 [file mmc2.docx]

***Editorial disclaimer:*** *This translation in Spanish was submitted by the authors and we reproduce it as supplied. It has not been peer reviewed. Our editorial processes have only been applied to the original abstract in English, which should serve as reference for this manuscript.*

**RESUMEN**

**Antecedentes:** La prevalencia de epilepsia en América Latina y el Caribe (ALC) se ha mantenido elevada durante los últimos 20 años. Se necesitan datos sobre la carga de epilepsia para la planificación de la atención médica y la asignación de recursos. Sin embargo, no se ha realizado un análisis sistemático de la carga de epilepsia en ALC.

**Métodos:** Se extrajeron datos de todos los países de ALC del estudio Global Burden of Disease (GBD) de 1990 a 2019. La carga de epilepsia se midió como prevalencia, mortalidad y años de vida ajustados por discapacidad (AVAD; definido por la suma de años de vidas perdidas [AVP] por mortalidad prematura y años vividos con discapacidad [AVD]), por edad, sexo, año y país. Se informaron números absolutos, tasas e intervalos de incertidumbre del 95%. Realizamos análisis correlacionales entre las métricas de carga y el índice sociodemográfico (SDI).

**Resultados:** La carga de la epilepsia disminuyó alrededor del 20% en ALC, liderada por la reducción de los AVP. En 2019, encontramos que 6·3 millones de personas vivían con epilepsia activa de cualquier causa (95% UI 5·3 - 7·4), con 3·22 millones (95% UI 2·21 - 4·03) y 3·11 millones (95% UI 2·21 a 4·03) de casos de epilepsia con etiología identificable y epilepsia idiopática, respectivamente. El número de AVAD representó el 9,51% (1·37 millones, 95% IU 0·99 - 1·86) de la carga global de epilepsia en 2019. La carga estandarizada por edad fue 175·9 por 100 000 habitantes (95% IU 119·4 - 253·3), con una tendencia de distribución de edad bimodal (más alta en jóvenes y ancianos) y fue impulsada por estimaciones altas de AVD. La carga fue mayor en hombres y adultos mayores, principalmente debido a los altos AVP y la mortalidad. El consumo de alcohol se asoció con el 17% de los AVAD informados. Las estimaciones de la IDE influyeron significativamente en esta carga (los países con un SDI alta tuvieron menos carga de epilepsia y mortalidad, pero no prevalencia o discapacidad).

**Interpretación:** La carga de epilepsia ha disminuido en ALC durante los últimos 30 años. Sin embargo, ALC todavía se posiciona como la tercera región con la mayor carga mundial de epilepsia. Esta reducción fue mayor en los niños, pero la carga y la mortalidad aumentaron en los adultos mayores. La carga de la epilepsia se basa en la discapacidad; sin embargo, las estimaciones relacionadas con la mortalidad son aún más elevadas que en otras regiones. El consumo de alcohol y el desarrollo de los países son determinantes importantes de esta carga. Existe una necesidad urgente de mejorar el acceso a la atención de epilepsia en ALC, en particular para los adultos mayores. En la región se debe priorizar el fortalecimiento de la atención primaria con herramientas de telemedicina y aprendizaje en línea y la promoción de la modificación de los factores de riesgo.

**Financiamiento:** Esta investigación fue autofinanciada por los autores.

**Palabras clave:** Carga de la enfermedad, epilepsia, epidemiología.
